# Olfactory memory is enhanced in mice exposed to extremely low-frequency electromagnetic fields via Wnt/β-catenin dependent modulation of subventricular zone neurogenesis

**DOI:** 10.1038/s41598-017-18676-1

**Published:** 2018-01-10

**Authors:** Alessia Mastrodonato, Saviana Antonella Barbati, Lucia Leone, Claudia Colussi, Katia Gironi, Marco Rinaudo, Roberto Piacentini, Christine A. Denny, Claudio Grassi

**Affiliations:** 1 Università Cattolica del Sacro Cuore, Institute of Human Physiology, Rome, 00168 Italy; 2 Columbia University, Department of Psychiatry, New York, NY 10032 USA; 3 Research Foundation for Mental Hygiene Inc. (RFMH), Division of Integrative Neuroscience, New York State Psychiatric Institute (NYSPI), New York, NY 10032 USA; 4 CNR, Institute of Cell Biology and Neurobiology, Monterotondo (RM), 00015 Italy; 5 Fondazione Policlinico Universitario A. Gemelli, Rome, 00168 Italy

## Abstract

Exposure to extremely low-frequency electromagnetic fields (ELFEF) influences the expression of key target genes controlling adult neurogenesis and modulates hippocampus-dependent memory. Here, we assayed whether ELFEF stimulation affects olfactory memory by modulating neurogenesis in the subventricular zone (SVZ) of the lateral ventricle, and investigated the underlying molecular mechanisms. We found that 30 days after the completion of an ELFEF stimulation protocol (1 mT; 50 Hz; 3.5 h/day for 12 days), mice showed enhanced olfactory memory and increased SVZ neurogenesis. These effects were associated with upregulated expression of mRNAs encoding for key regulators of adult neurogenesis and were mainly dependent on the activation of the Wnt pathway. Indeed, ELFEF stimulation increased Wnt3 mRNA expression and nuclear localization of its downstream target β-catenin. Conversely, inhibition of Wnt3 by Dkk-1 prevented ELFEF-induced upregulation of neurogenic genes and abolished ELFEF’s effects on olfactory memory. Collectively, our findings suggest that ELFEF stimulation increases olfactory memory via enhanced Wnt/β-catenin signaling in the SVZ and point to ELFEF as a promising tool for enhancing SVZ neurogenesis and olfactory function.

## Introduction

The SVZ of the lateral ventricle and the subgranular zone of the dentate gyrus (DG) are the two main regions of the adult mammalian brain where neurogenesis is maintained throughout life^1^. In these neurogenic niches, neural stem/precursor cells (NSCs) proliferate and differentiate into migrating neuroblasts that generate new neurons integrating into pre-existing circuits^2,3^. Extensive studies have shown that numerous stimuli, including exercise, enriched environment and genetic strategies, positively affect adult neurogenesis^4^. Among these, extremely low-frequency electromagnetic fields (ELFEF) have been shown to promote NSC proliferation and their neuronal differentiation^5–7^. Our previous studies demonstrated that ELFEF increase adult hippocampal neurogenesis and enhance the survival of newborn neurons^8–10^. These newly generated granule neurons functionally integrate into the DG, increase synaptic plasticity, and improve hippocampus-dependent memory^8,9^.

Recent studies revealed that adult SVZ neurogenesis is necessary for cognitive functions such as perceptual learning and olfactory memory^11,12^. However, the functional impact of these newly-formed neurons on olfactory memory is still hotly debated and limited information is available on the mechanisms and signaling pathways involved. Furthermore, whether ELFEF stimulation may improve SVZ neurogenesis and olfactory memory is, at present, unknown.

Adult neurogenesis is regulated by specific gene expression cascades. In particular, the canonical Wnt/β-catenin signaling pathway plays a pivotal role in the regulation of cell proliferation, differentiation, migration, genetic stability, and apoptosis, as well as in the maintenance of adult stem cells in a pluripotent state^13^. In the presence of an extracellular Wnt ligand, the intracellular levels of the transcription factor β-catenin increase, allowing its migration to the nucleus where it binds to T-cell factor/lymphoid enhancer-binding factor (TCF/LEF), thereby activating the expression of Wnt target genes including NeuroD1^14^. In the absence of a Wnt ligand, β-catenin is phosphorylated for ubiquitin-proteasome-mediated degradation, thus switching off the expression of Wnt target genes^13^. Numerous reports have shown that Wnt/β-catenin pathway is important for both hippocampal and SVZ neurogenesis^15^ and that age and activity may respectively decrease and increase adult neurogenesis via antagonists of the Wnt signaling pathway^16^. Since adult neurogenesis and olfactory function decline with aging^17^, there is considerable interest in developing strategies to promote adult olfactory neurogenesis and improve olfactory memory.

Here, we investigated the impact of ELFEF stimulation on olfactory memory and SVZ neurogenesis by focusing on the role played by Wnt/β-catenin signaling in these effects. We show that mice exposed to ELFEF exhibit increased odor discrimination and improved short- and long-term olfactory memory, that are associated with enhanced adult neurogenesis in the SVZ and olfactory bulb (OB). The improved olfactory learning and memory is dependent on Wnt3 signaling to increase nuclear localization of the transcription factor β-catenin, and the expression of genes orchestrating NSC proliferation and neuronal cell-fate specification including Hes1 and Mash1.

## Results

### ELFEF exposure increases odor discrimination and olfactory memory

**Figure 1:**
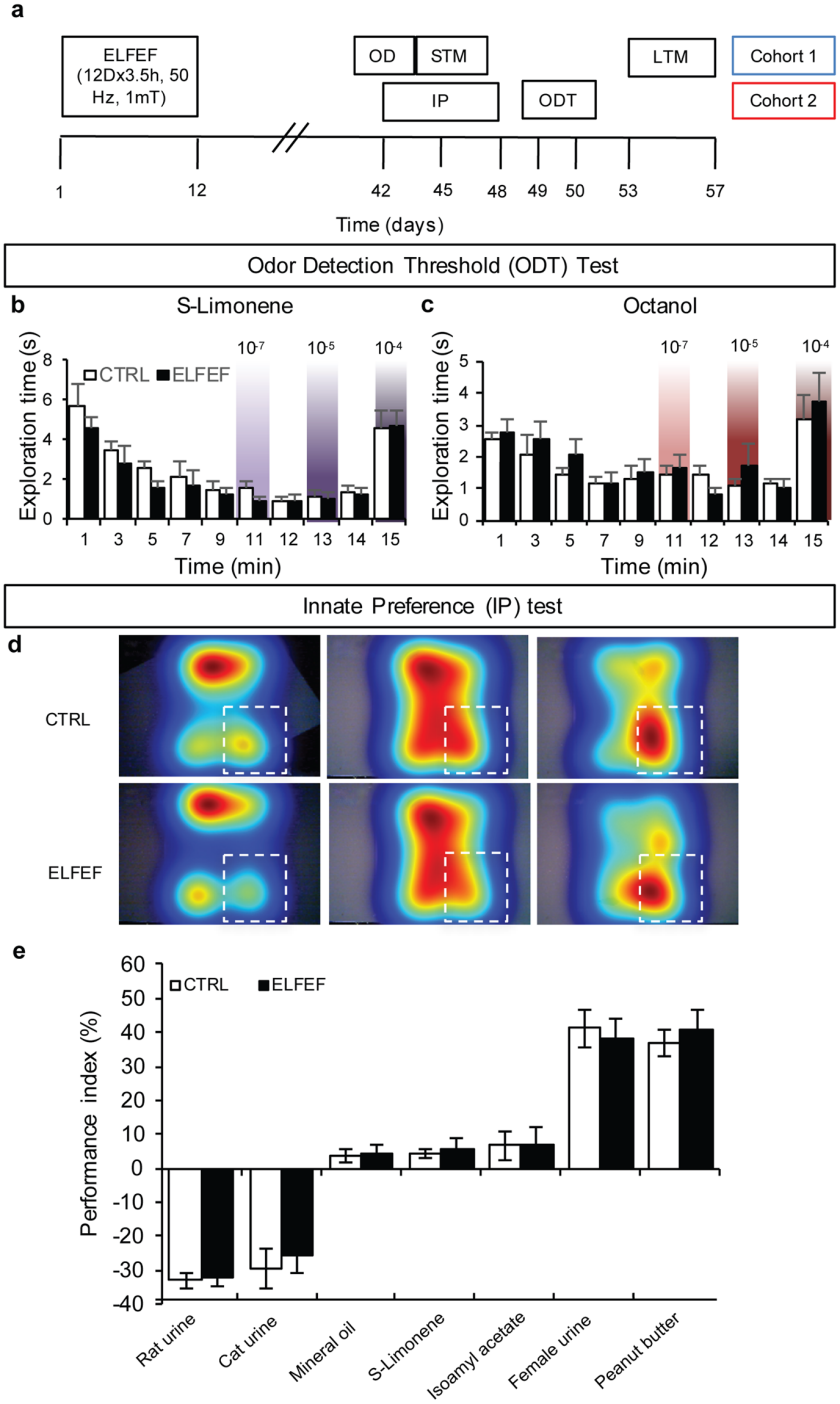
ELFEF does not alter odor detection threshold or innate preference. (**a**) Timeline of experiments. (**b**,**c**) Bar graphs showing time spent exploring cotton sticks soaked in mineral oil (m.o., white area) or diluted odorant (colored area) for two different odorants, (S)-Limonene and Octanol. Control and ELFEF-exposed mice did not differ from each other. (**d**) Innate preference test heat maps (controls: upper panels; ELFEF-exposed mice: lower panels) showing time spent in different zones of the behavioral apparatus (color code: blue, low; red, high). The white dotted squares indicate the quadrant where the tested odor was placed. (**e**) Bar graphs showing preference indexes in the innate preference test in response to an aversive (rat or cat urine), neutral (mineral oil, S-Limonene, Isoamyl acetate), or pleasant odor (female mice urine or peanut butter). Considering the percentage of time spent in one quadrant as P, the preference index was calculated as: (P – 25)/0.25. Control and ELFEF-exposed mice did not differ from each other. Values are expressed as means ± SEM (n = 7–10 mice per group). OD: odor discrimination test; STM: short-term memory; LTM: long-term memory.

Prior to investigating the effects of ELFEF on olfactory memory, we performed odor detection threshold and innate preference tests to exclude possible confounding effects of ELFEF-induced changes in either odorant perception or innate odor-driven behavior. Information gained from these experiments also allowed us to identify the optimal odor concentrations to be used for olfactory memory tests. Figure 1a shows the timeline of experiments described below.

In the threshold detection paradigm, we presented cotton sticks soaked in mineral oil to the animal, followed by cotton sticks soaked with increasing concentrations of a specific odorant diluted in mineral oil. The presentation eliciting higher exploration time compared to the previous mineral oil exposure was considered to be the concentration threshold for the odorant. Both control and ELFEF-exposed mice showed a significant increase in the exploration time, compared with mineral oil, at the concentration of 10^−4^%, for the two tested odorants (for Octanol: n = 9 mice for each group, p = 0.015 for control and p = 0.012 for ELFEF-exposed mice; for (S)-Limonene: n = 7 for each group, p = 0.003 for control and p = 0.0006 for ELFEF-exposed mice, paired Student’s *t-*test) and no differences were found between groups. Indeed RM ANOVA for Octanol revealed no effect of Group (F_(1,16)_ = 0.540, p = 0.473), a significant effect of Time (F_(1,2)_ = 11.160, p = 0.0002) and no interaction (Group × Time, F_(2,32)_ = 0.830, p = 0.921). Likewise, for (S)-Limonene there was no effect of Group (F_(1,12)_ = 0.149, p = 0.706), a significant effect of Time (F_(1,2)_ = 37.341, p < 0.0001) and no interaction (Group × Time, F_(2,24)_ = 0.356, p = 0.704, Fig. 1b,c). These findings suggest that odor detection threshold was not affected in mice exposed to ELFEF.

To test whether ELFEF stimulation influenced the innate response to odors, we used a protocol modified from Root and colleagues^18^ (Fig. 1d). In the absence of odor, mice explored the chamber without preference for any quadrant and the total distances traveled as well as the time spent in the center of the arena by control- and ELFEF-stimulated animals were similar (Supplementary Fig. S1), thus excluding any difference in locomotor activity between the two groups. Addition of the odor in one quadrant resulted in different behavioral responses: female urine and peanut butter elicited attraction (performance index = 41.13 ± 5.83 and 36.97 ± 4.08 for control mice, and 37.89 ± 5.83 and 41.02 ± 5.88 for ELFEF-exposed mice, respectively; Fig. 1d,e); whereas rat urine and cat urine elicited aversion (performance index = −32.95 ± 2.33 and −29.58 ± 6.14 for control mice and −31.85 ± 2.67 and −25.51 ± 5.30 for ELFEF-exposed mice, respectively). Mineral oil, Isoamyl acetate and (S)-Limonene were neutral in this assay (performance index = 2.29 to 6.65). Using a RM ANOVA for all odors, we found no effect of Group (F_(1,12)_ = 0.642, p = 0.438). Although there was an effect of the Odor (F_(1,6)_ = 90.260, p < 0.001), there was no interaction (Group × Odor, F_(6,72)_ = 0.330, p = 0.918).

**Figure 2:**
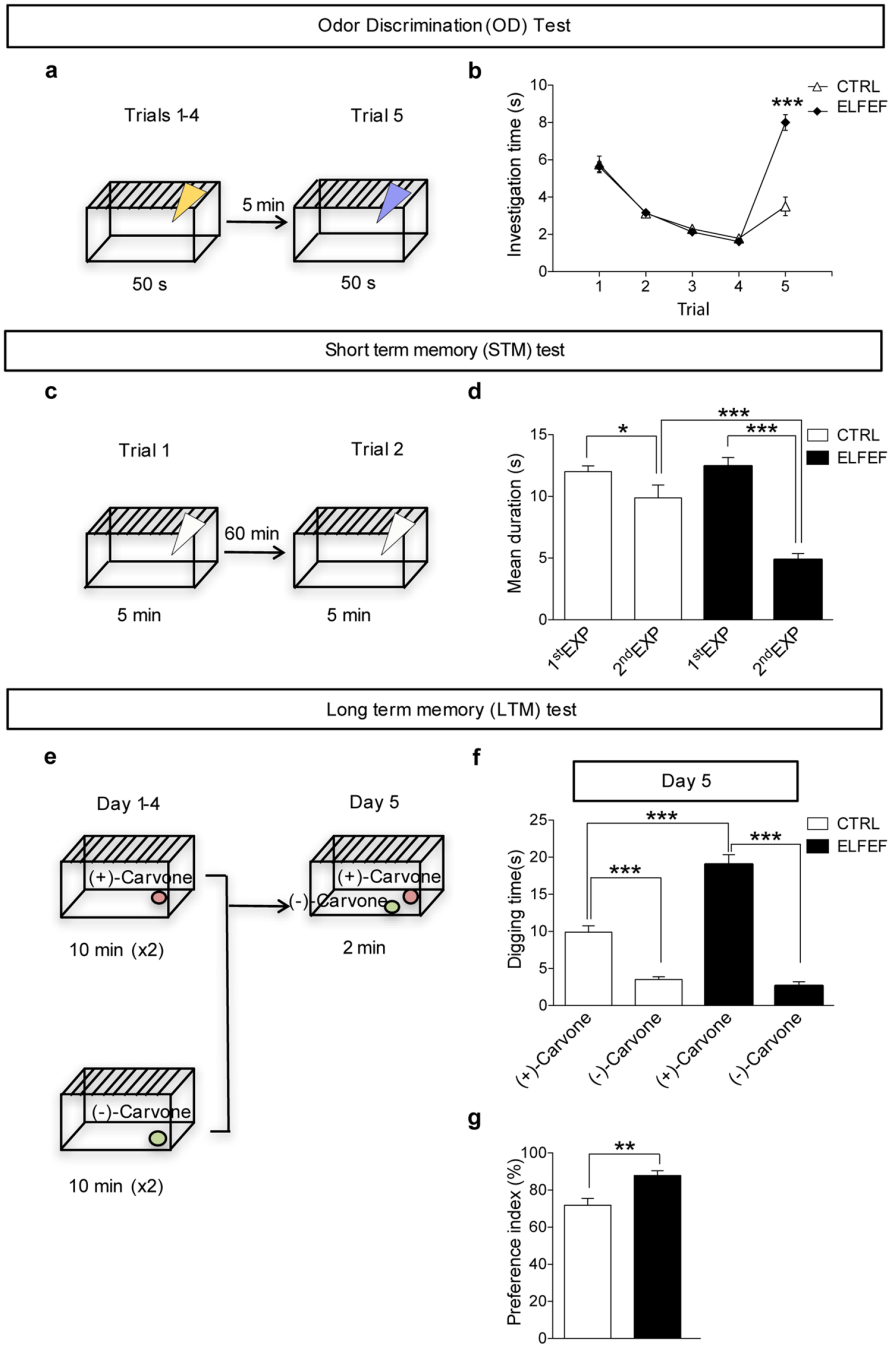
ELFEF exposure results in improved odor memory. (**a**) Experimental design for odor discrimination test. (**b**) Both control and ELFEF-exposed mice similarly decreased the investigation time during habituation. However, ELFEF-exposed mice showed a significantly higher discrimination between the familiar and a novel odor when compared with controls. (**c**) Experimental design for short-term memory test. (**d**) Each bar represents the mean time spent investigating the odor on the first and the second exposures. ELFEF-exposed mice showed a shorter investigation time on the second presentation than control. (**e**) Experimental design for long-term memory test. (**f**) In both conditions, mice were able to recognize the reward-associated odor but ELFEF-exposed mice spent a significantly longer digging time near the odor that had been previously rewarded when compared to controls, as shown by the mean digging time (**f**) and by preference index values (**g**). Values are expressed as means ± SEM. (n = 7–8 mice per group). *p < 0.05, **p < 0.01, ***p < 0.001.

A different cohort of mice was then tested in the odor discrimination, short and long-term memory tasks. In the odor discrimination paradigm, mice must discriminate a novel odor from a familiar odor (Fig. 2a). During the 4 habituation trials, control and ELFEF-exposed mice spent a comparable amount of time investigating the odor. A RM ANOVA revealed no effect of Group (F_(1,14)_ = 0.217, p = 0.649), but an effect of Time (F_(1,3)_ = 137.235, p < 0.0001). Moreover, there was no interaction (Group × Time, F_(3,42)_ = 0.111, p = 0.953). On the dishabituation trial both groups were able to discriminate the novel odor from the familiar one (n = 8 mice per group, p = 0.0001 for control and p = 0.007 for ELFEF-exposed mice, paired Student’s *t*-test). However, during trial 5, ELFEF-exposed mice spent a significantly greater amount of time investigating the novel odor when compared with control mice (8.0 ± 0.5 *vs*. 3.5 ± 0.5 s, respectively; n = 8 mice per group; p = 0.00001 unpaired Student’s *t-*test; Fig. 2b).

We next exposed mice to a short-term odor memory test (Fig. 2c). In this paradigm, mice must remember a previously encountered odor for 60 minutes following the first presentation. Control and ELFEF-exposed mice spent a comparable amount of time investigating the odor during the first presentation (12.4 ± 0.8 *vs*. 12.0 ± 0.5 s, respectively; Fig. 2d). However, ELFEF-exposed mice showed a more marked drop in exploration time on the second presentation of the odor than control mice (4.9 ± 0.5 s [n = 7] and 9.9 ± 1.1 s [n = 8], respectively; p = 0.001, unpaired Student’s *t-*test), indicating improved short-term memory.

To assess the impact of ELFEF on long-term odor memory, we habituated mice for 4 days to associate one of two related odors (enantiomers) with a sugar reward placed below bedding (Fig. 2e). Twenty-four hours after the completion of the habituation protocol, we tested the ability of mice to remember which odor was associated with the sugar reward by measuring the time spent digging for each odorant. Both control and ELFEF-exposed mice spent more time near the odor previously associated with a reward, but ELFEF-exposed mice showed a significantly longer digging time near the odor previously rewarded than control mice. Digging times near the rewarded odor [(+)-Carvone] were 9.9 ± 0.9 and 19.0 ± 1.4 s for control and ELFEF-exposed mice, respectively (n = 8 mice per group; p = 0.00006 unpaired Student’s *t-*test), whereas those near the non-rewarded odor [(−)-Carvone] were 3.5 ± 0.4 and 2.6 ± 0.9 s, respectively (p = 0.2 unpaired Student’s *t-*test). Multiple comparisons carried out by two-way ANOVA and Bonferroni post-hoc test revealed significant differences (p < 0.001 for all comparisons; Fig. 2f). Moreover, ELFEF-exposed mice had a higher preference index for the rewarded odor than control mice (87.9 ± 2.8% for ELFEF-exposed mice *vs*. 71.9 ± 3.9% for control mice; p = 0.003 unpaired Student’s *t-*test; Fig. 2g). These data indicate that acquisition and retention of long-term odor-associated memory are improved following ELFEF stimulation.

To rule out any dependence of the observed effects on the specific tested odor we repeated this set of experiments in a different cohort of mice using different couples of odors. Specifically, animals were tested in the: i) odor discrimination paradigm using the (+)-Carvone and (−)-Carvone enantiomers; ii) short-term memory test using Octanol; and iii) long-term memory test using Linalool and Phenyl acetate. In the odor discrimination, during the 4 habituation trials, control and ELFEF-exposed mice spent a comparable amount of time investigating the odor. A RM ANOVA revealed no effect of Group (F_(1,12)_ = 3.725, p = 0.078), but an effect of Time (F_(1,3)_ = 25.623, p < 0.0001). Moreover, there was no interaction (Group × Time, F_(3,36)_ = 1.327, p = 0.280). On the dishabituation trial both groups were able to discriminate the novel odor from the familiar one (n = 7 mice per group, p = 0.0006 for control and p = 0.003 for ELFEF-exposed mice, paired Student’s *t*-test). However, during trial 5, ELFEF-exposed mice spent a significantly greater amount of time investigating the novel odor when compared with control mice (5.3 ± 2.4 *vs*. 2.9 ± 0.7 s, respectively; p = 0.025 unpaired Student’s *t*-test). In the short-term memory, ELFEF-mice explored significantly less time compared to control mice on the second presentation (3.2 ± 0.3 s [n = 8] *vs*. 6.2 ± 0.4 s [n = 6], respectively; p = 0.00005 unpaired Student’s *t-*test). In the long-term memory test, both control and ELFEF-exposed mice spent more time near the odor previously associated with a reward, but ELFEF-exposed mice showed a significantly longer digging time near the odor previously rewarded than control mice. Digging times near the rewarded odor (Linalool) were 7.1 ± 2.1 and 16.6 ± 5.5 s for control and ELFEF-exposed mice, respectively (n = 7 mice per group; p = 0.001 unpaired Student’s *t-*test), whereas those near the non-rewarded odor (Phenyl acetate) were 3.2 ± 1.9 and 2.9 ± 1.9 s, respectively (p = 0.847 unpaired Student’s *t-*test). Furthermore, we analyzed the sniffing time of (R)-Limonene, (S)-Limonene and Isoamyl acetate and the digging time of (−)-Carvone in 50 s bins, comparing them across the different behavioral tests (Supplementary Fig. S2a–d). RM ANOVA for all odors revealed no effect of Group (F_(1,13)_ = 0.050, p = 0.826) or Odor (F_(1,3)_ = 0.364, p = 0.779) and no interaction (Group × Odor, F_(3,39)_ = 0.735, p = 0.537). Collectively, these data suggest that ELFEF stimulation increases odor discrimination and olfactory memory, and these effects are not dependent on a specific odor.

### ELFEF exposure increases SVZ neurogenesis

**Figure 3:**
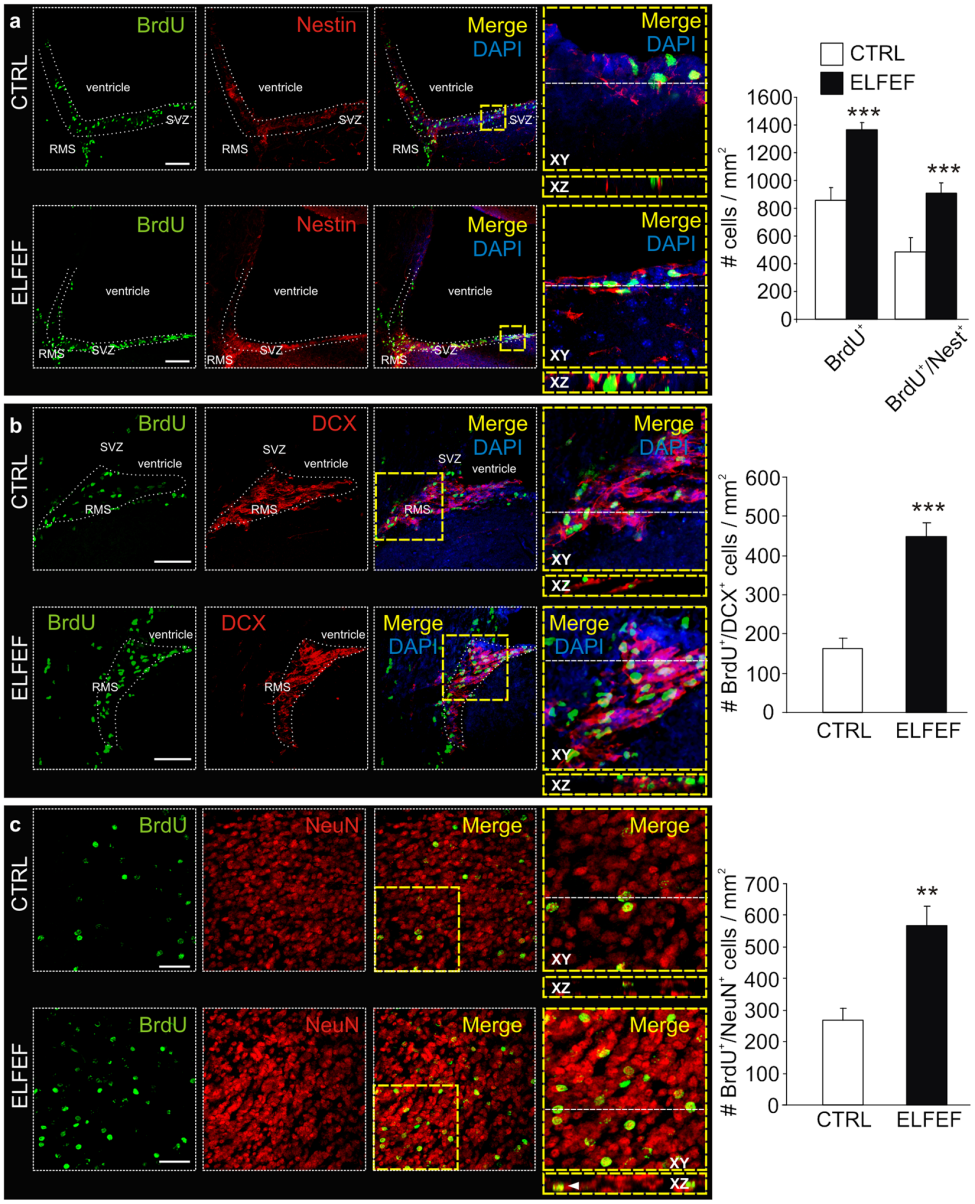
ELFEF exposure increases NSC proliferation in the SVZ and integration of newborn neurons in the olfactory bulb. Immunohistochemical analyses performed in coronal (panels a and b) and sagittal (panel c) brain sections. When compared with controls, the ELFEF-exposed (12D × 3.5 h) mice exibited increased numbers of: (**a**) BrdU^+^ and BrdU^+^/Nestin^+^ double-labeled cells in the SVZ; (**b**) newborn NSCs differentiating towards the neuronal phenotype (i.e., BrdU^+^/DCX^+^ double-labeled cells) in the RMS, and (**c**) mature neurons (i.e., BrdU^+^/NeuN^+^ double-labeled cells) in the OB. Right panels in each row show enlargements of dotted yellow boxes outlined in the respective “merged” images. Images on the bottom of each enlargement represent XZ cross section, corresponding to the upper dotted white lines, from confocal Z-stacks carried out to study co-localization. Scale bar: 100 µm for panels a and b, 50 µm for panels c. Bar graphs on the right show the number of cells/mm^2^ for each condition studied. Values are expressed as means ± SEM. **p < 0.01, ***p < 0.001 *vs*. CTRL.

Experimental evidence suggests that adult SVZ neurogenesis plays a critical role in odor discrimination and olfactory memory^11,12,19^. Therefore, we performed immunohistochemical experiments to assess whether increased SVZ neurogenesis correlated with the ELFEF-induced enhancement of odor memory. ELFEF-exposed mice exhibited enhanced NSC proliferation in the SVZ, as assessed by the number of BrdU^+^ cells that increased from 856 ± 93 /mm^2^ in one hemisphere of control mice (n = 6) to 1365 ± 102/mm^2^ in ELFEF-exposed mice (n = 6; +60%; p = 0.0009, Mann-Whitney test, Fig. 3a). A similar increase was also observed for cells expressing Ki67, a protein strictly associated with cell proliferation. The number of Ki67^+^ cells raised from 1581 ± 184/mm^2^ in controls (n = 3) to 2150 ± 177/mm^2^ in ELFEF-exposed mice (n = 3) (+36%; p = 0.050, Mann-Whitney test). Approximately half of BrdU^+^ cells (42–51%) also expressed Nestin, a marker of progenitor cells; the number of BrdU^+^/Nestin^+^ cells was 484 ± 53 and 907 ± 75/mm^2^ in control and ELFEF-exposed animals, respectively (+88%, p = 0.0002, Mann-Whitney test; Fig. 3a). The number of newborn NSCs differentiating towards the neuronal phenotype (BrdU^+^/DCX^+^ cells) was similarly increased in the rostral migratory stream (RMS) of ELFEF-exposed mice when compared with control mice (448 ± 35 [n = 6] *vs*. 162 ± 28 [n = 6] cells/mm^2^; p = 0.0002, Mann-Whitney test; Fig. 3b). Accordingly, the number of newborn cells becoming mature neurons in the OB (i.e., BrdU^+^/NeuN^+^ cells) 30 days after the end of ELFEF stimulation protocol was increased in ELFEF-exposed mice when compared with controls (569 ± 61 [n = 4] *vs*. 268 ± 37 [n = 3] cells/mm^2^; p = 0.004, Mann-Whitney test; Fig. 3c). The BrdU^+^/NeuN^+^ double-labelled cells in the OB were the vast majority (82.8 ± 4.6%) of total BrdU^+^ cells.

### The ELFEF-induced increase of SVZ neurogenesis relies on Wnt/β-catenin signaling

**Figure 4:**
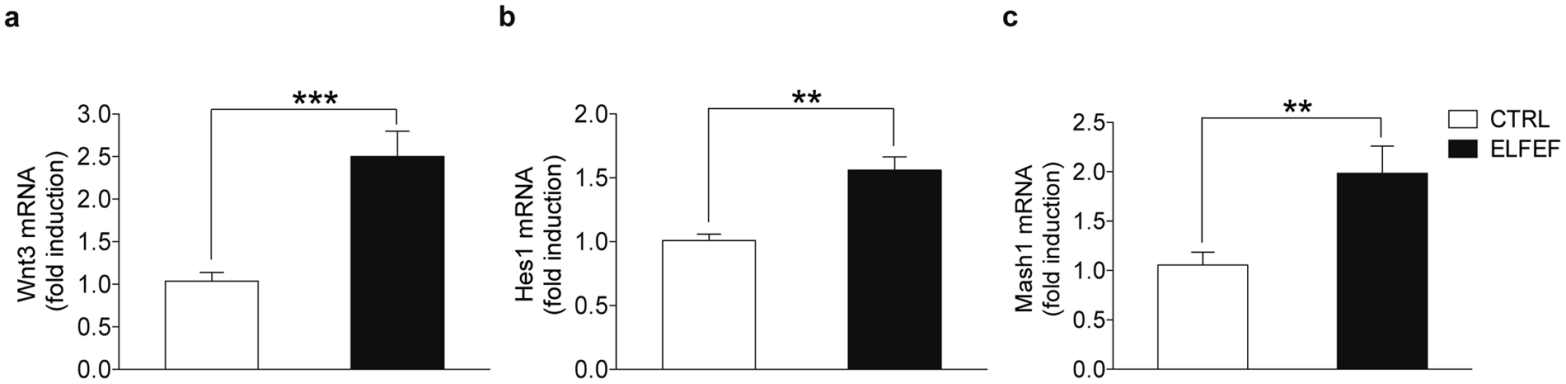
ELFEF stimulation increases the expression of neurogenic genes in the SVZ. Bar graphs showing levels of mRNA encoding for Wnt3 (**a**), Hes1 (**b**), and Mash1 (**c**) in control and ELFEF-exposed (4D × 3.5 h) mice. Values are expressed as means ± SEM of fold increase in the ratio of each gene/TBP, with the value of control taken as 1.0 (n = 3 mice per group). **p < 0.01, ***p < 0.001.

To determine the molecular mechanisms underlying the ELFEF-induced increases in NSC proliferation and neuronal differentiation, we studied the expression of neurogenic genes in the SVZ after ELFEF stimulation (Fig. 4). Specifically, we used RT-qPCR to measure mRNA levels of Wnt3, a signal molecule playing pivotal roles in various facets of adult neurogenesis^13^, as well as those of Hes1 and Tlx, master regulators of NSC self-renewal^20,21^, and those of Mash1, Pax6 and Prox1, as transcriptional factors involved in fate determination^22–24^. We found increased levels of Wnt3 (+150.0%, p = 0.0003 unpaired Student’s *t-*test; Fig. 4a), Hes1 (+40.7%, p = 0.0014 unpaired Student’s *t-*test; Fig. 4b), and Mash1 (+96.0%, p = 0.0048 Mann Whitney test; Fig. 4c) in SVZ extracts from ELFEF-exposed mice when compared with control mice. Of note, mRNA levels of other neurogenic transcriptional regulators of SVZ neurogenesis, such as Tlx, Pax6, and Prox1 were unchanged in the above conditions (Supplementary Fig. S3). Parallel experiments performed on hippocampal extracts of the same cohorts of animals revealed that, while the mRNA level of the neurogenic genes Hes1 and Mash1 was upregulated as previously reported^9^, Wnt3 mRNA levels were unchanged (Supplementary Fig. S3d–f).

**Figure 5:**
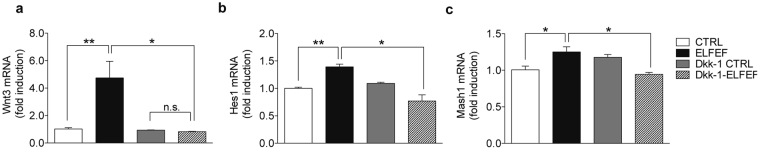
ELFEF stimulation enhances the expression of neurogenic genes in cultured NSCs isolated from the SVZ. Bar graphs showing levels of mRNA encoding for Wnt3 (**a**), Hes1 (**b**), and Mash1 (**c**) in control and ELFEF-exposed NSCs cultured in the absence or the presence of Dkk-1. ELFEF exposure increased expression for these mRNAs, but this increase was blocked when NSCs were cultured in the presence of Dkk-1 (100 ng/mL). Values are expressed as means ± SEM of the fold increase in the ratio of each gene/TBP, with the value of control (unexposed) NSCs taken as 1.0. Experiments were performed in triplicate. n.s. = not significant, *p < 0.05, **p < 0.01.

To further investigate the molecular mechanisms underlying ELFEF’s effects, we extended our studies to an *in vitro* model of NSCs isolated from the SVZ of newborn mice. RT-qPCR analysis performed after 24 hour-exposure to ELFEF showed a very marked increase of Wnt3 mRNA (+374%; p = 0.002 Mann Whitney test; Fig. 5a), that was accompanied by significant increases in Hes1 (+39.3%; p = 0.002 Mann Whitney test; Fig. 5b), and Mash1 mRNA (+71%; p = 0.026 Mann Whitney test; Fig. 5c) when compared to control NSCs. The blockade of Wnt signaling by treatment of NSC cultures with Dkk-1 (100 ng/mL for 24 hours) prevented the ELFEF-induced increases of Wnt3 (p = 0.024 Mann Whitney test; Fig. 5a), Hes1 (p = 0.024 Mann Whitney test; Fig. 5b), and Mash1 (p = 0.024 Mann Whitney test; Fig. 5c) mRNA. Similarly to what we observed *in vivo*, ELFEF failed to change mRNA levels of other transcriptional regulators of SVZ neurogenesis, including Pax6 (Supplementary Fig. S4a) and Prox1 (Supplementary Fig. S4b), that are fate determinants for OB interneurons and oligodendrocytes respectively, and Tlx (Supplementary Fig. S4c), which is exclusively expressed in astrocyte-like B cells of the SVZ-OB system^21,23,24^.

**Figure 6:**
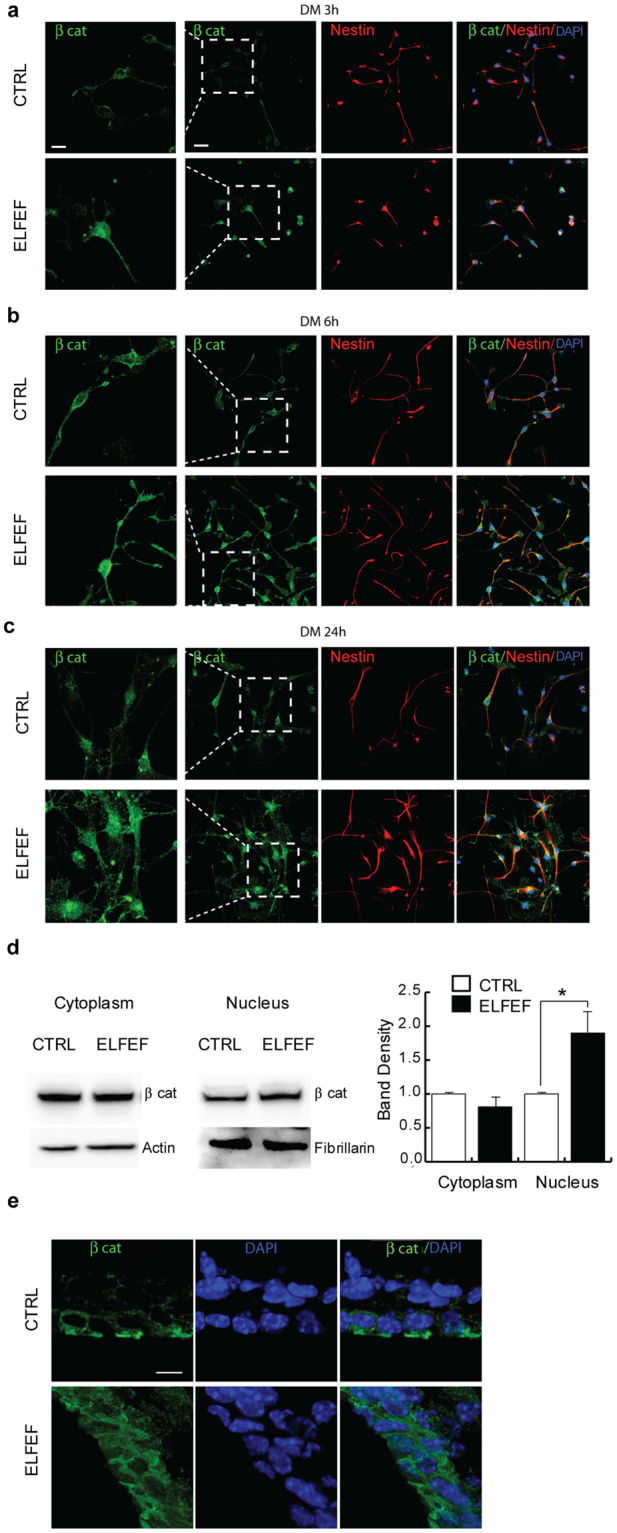
ELFEF stimulation increases β-catenin nuclear accumulation in cultured NSCs isolated from the SVZ upon differentiation. (**a**–**c**) Representative confocal images showing β-catenin localization (green) in control- and ELFEF-exposed NSCs at 3 h (**a**), 6 h (**b**), and 24 h (**c**) after the induction of differentiation (DM). Higher magnifications are provided in the first column (scale bar: 10 µm). Cells were counterstained with Nestin (red), and nuclei were stained with DAPI (blue) (scale bar: 25 µm). (**d**) Western blot showing different distribution of β-catenin in nuclear and cytoplasmic fractions from NSCs induced to differentiate for 24 h. Bar graph shows band density analysis. ELFEF exposure increased β-catenin in the nuclear fraction, but not in the cytoplasmic fraction. Full length blots are presented in Supplementary Fig. S5. (**e**) Representative images showing β-catenin localization in NSCs from the SVZ in control and ELFEF mice. Nuclei were counterstained with DAPI (scale bar: 10 µm). Values are expressed as means ± SEM. (n = 3 per group). *p < 0.05.

We also tested the hypothesis that ELFEF-dependent upregulation of Wnt3 was paralleled by the activation of its downstream transcription factor β-catenin. Indeed, NSCs exposed to ELFEF exhibited an anticipated and longer lasting expression of β-catenin in the nucleus compared to control NSCs, as shown by confocal analyses performed at 3, 6 and 24 h of differentiation (Fig. 6a–c). Analysis of nuclear and cytoplasmic fractions by western blot confirmed the increased levels of β-catenin in the nuclei of ELFEF-exposed NSCs (Fig. 6d, p = 0.044 unpaired Student’s *t-*test; Supplementary Fig. S5). Notably, confocal analysis revealed an increased nuclear β-catenin signal also in the SVZ NSCs of ELFEF-exposed mice compared to control mice (Fig. 6e).

### Inhibition of Wnt/β-catenin signaling abolishes ELFEF-induced increases in SVZ neurogenesis and olfactory memory

**Figure 7:**
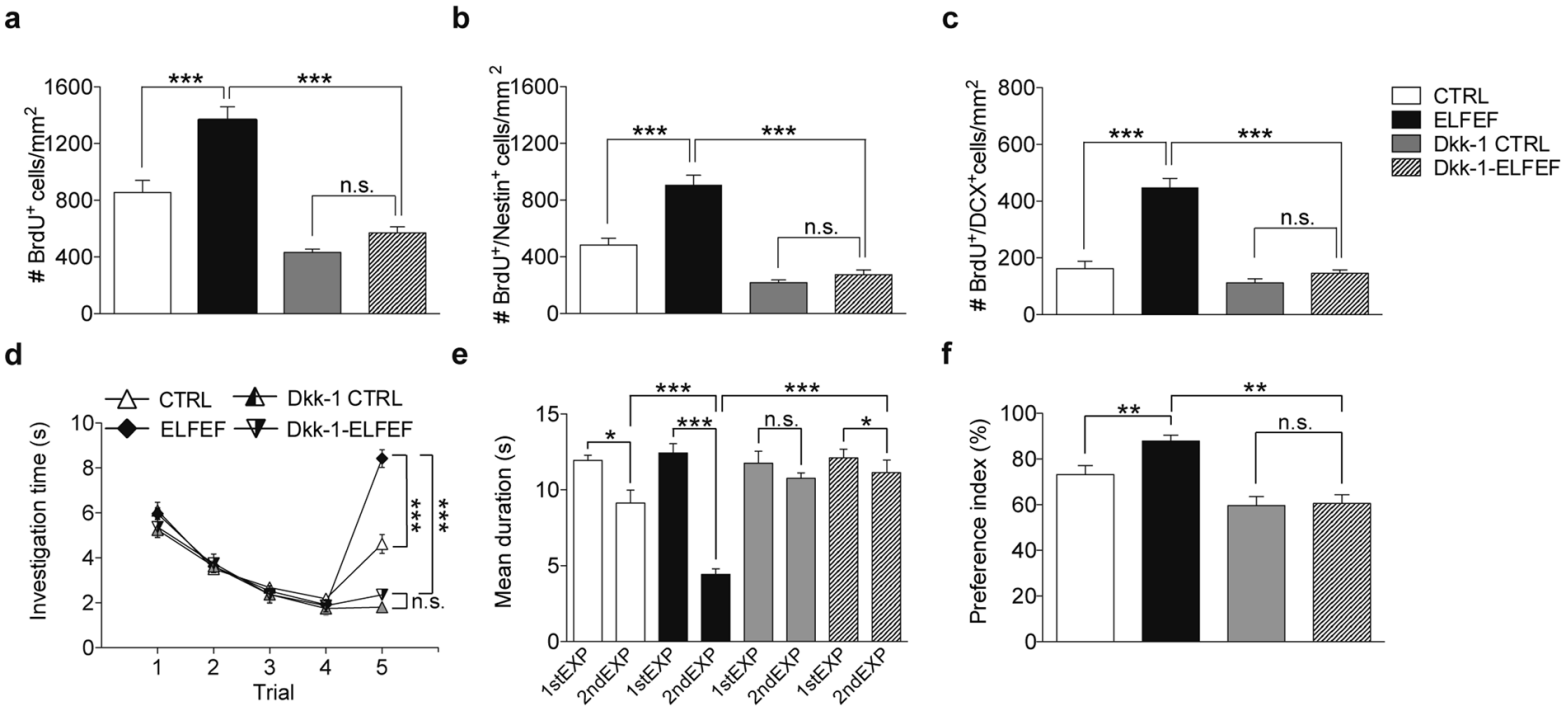
Inhibition of Wnt/β-catenin signaling impairs ELFEF-induced increases of SVZ neurogenesis and olfactory memory. (**a**–**c**) Bar graphs showing the number of BrdU^+^ (**a**) and BrdU^+^/Nestin^+^ (**b**) cells in the SVZ, and BrdU^+^/DCX^+^ cells (**c**) in the RMS of control and ELFEF-exposed mice perfused with either vehicle (CTRL) or Dkk-1. (**d**) In the odor discrimination test, ELFEF-exposed mice injected with vehicle (ELFEF) showed a higher discrimination ability when compared to ELFEF-exposed mice injected with Dkk-1 (Dkk-1-ELFEF). (**e**) Injection of Dkk-1 into the SVZ abolished the enhancement of olfactory memory induced by ELFEF. (**f**) In the long-term olfactory memory tests, the preference index of Dkk-1-ELFEF mice was significantly less when compared to ELFEF mice. Values are expressed as means ± SEM. (n = 7–8 mice per group). n.s. = not significant, *p < 0.05, **p < 0.01, ***p < 0.001.

Collectively, data reported above suggest Wnt upregulation is the major determinant of the ELFEF-induced increase in SVZ neurogenesis. To validate these findings *in vivo*, we investigated the effects of the Wnt inhibitor, Dkk-1, on the ELFEF-induced modulation of SVZ neurogenesis and memory in mice. In particular, Dkk-1 (200 ng/0.5 µL) was stereotaxically injected (at the rate of 0.1 µL/min) into the lateral ventricle of control and ELFEF-exposed mice for 12 days. We found that Dkk-1 prevented the ELFEF-induced increases in proliferation and neuronal differentiation of SVZ NSCs. Indeed, the number of BrdU^+^, BrdU^+^/Nestin^+^ and BrdU^+^/DCX^+^ cells/mm^2^ in controls and ELFEF-exposed mice injected with Dkk-1 (n = 4 for both conditions) was: 432 ± 20 *vs*. 570 ± 39 (p = 0.695, Mann-Whitney test; Fig. 7a), 219 ± 20 *vs*. 274 ± 39 (p = 0.912, Mann-Whitney test; Fig. 7b) and 112 ± 16 *vs*. 145 ± 14 (p = 0.853, Mann-Whitney test; Fig. 7c), respectively. Comparisons between sham-stimulated mice treated with and without Dkk-1 revealed that the Wnt inhibitor significantly reduced the number of proliferating NSCs (p = 0.010, Mann-Whitney test; Fig. 7a).

We also tested whether intra-ventricular administration of Dkk-1 abolished the positive effects of ELFEF on odor discrimination and olfactory memory (Fig. 7d–f).

During the odor discrimination paradigm, all groups habituated to the odor at comparable levels: Group (F_(3,28)_ = 0.715, p = 0.551); Time (F_(3,3)_ = 171.887, p < 0.0001); Group × Time (F_(9,84)_ = 0.724, p = 0.685; RM ANOVA; Fig. 7d). However, during trial 5, ELFEF-exposed mice injected with Dkk-1 spent a significantly lower amount of time investigating the novel odor than ELFEF-exposed mice injected with vehicle (2.0 ± 0.2 *vs*. 8.1 ± 0.4 s, respectively; n = 8 per group; p = 9.7 × 10^−10^ unpaired Student’s *t-*test; Fig. 7d). Dkk-1 injection alone impaired odor discrimination (investigation time in the last habituation trial and in the dishabituation trial were: 1.4 ± 0.3 *vs*. 1.4 ± 0.2 s, respectively; p = 0.826 paired Student’s *t-*test; Fig. 7d), in compliance with a role of Wnt signaling in olfaction^25^.

In the short-term olfactory memory test Dkk-1 treatment similarly hindered ELFEF’s effects. In particular, ELFEF-exposed mice injected with Dkk-1 showed a significantly higher investigation time on the second odor exposure when compared to ELFEF-exposed mice injected with vehicle (11.1 ± 0.8 s [n = 8] *vs*. 4.4 ± 0.4 s [n = 7], respectively, p = 5.8 × 10^−6^, unpaired Student’s *t-*test; Fig. 7e). RM ANOVA analysis revealed an effect of Group (F_(3,27)_ = 7.725, p = 0.0007) and Time (F_(3,1)_ = 69.321, p < 0.0001) and a significant interaction Time × Group (F_(3,27)_ = 17.437, p < 0.0001). Consistent with a role of Wnt signaling in memory formation^25^, Dkk-1 injection in control mice impaired short-term odor memory (investigation time on first and second odor exposures were 11.8 ± 0.9 and 10.8 ± 0.4 s, respectively; n = 8; p = 0.316, paired Student’s *t-*test). In the long-term odor memory test, ANOVA analysis performed on all groups revealed a significant effect of Group (F_(3,27)_ = 14.889, p < 0.0001) and, according to the result obtained in the short-term memory test, we found that the performance of Dkk-1-injected mice exposed to ELFEF was significantly impaired compared to that of vehicle-injected mice exposed to ELFEF (preference index: 60.6 ± 4.1% [n = 7] *vs*. 88.1 ± 1.8% [n = 8], respectively; p = 0.00001 unpaired Student’s *t-*test; Fig. 7f). In control mice Dkk-1 infusion alone inhibited long term memory (digging time near the rewarded and not rewarded odors were: 2.5 ± 0.4 *vs*. 3.6 ± 0.4 s, respectively p = 0.065, paired Student’s *t-*test).

The correct placement of cannula used for Dkk-1 injection was routinely checked (Supplementary Fig. S6) and control experiments were performed to verify the effectiveness of Dkk-1 treatment and its spreading within the brain by measuring the Wnt downstream target β-catenin in both SVZ and hippocampus (Supplementary Fig. S7). Confocal microscopy and western blot analyses revealed that Dkk-1 injection into the SVZ reduced β-catenin protein expression in the SVZ (Supplementary Fig. S7a,c). Interestingly, no reduction of β-catenin was observed in the hippocampus of Dkk-1 treated mice (Supplementary Fig. S7b,c), suggesting that the drug selectively targeted Wnt signaling in the SVZ.

## Discussion

In this study, through a combination of *in vivo* and *in vitro* experiments, we found that exposure to ELFEF enhances olfactory learning and memory in mice through upregulation of Wnt/β-catenin signaling, which increases SVZ neurogenesis via enhanced expression of the neurogenic genes Hes1 and Mash1. Notably, inhibition of Wnt signaling abolished the ELFEF-induced upregulation of both neurogenic gene expression and olfactory memory.

Several studies have demonstrated that electromagnetic field stimulation exerts beneficial effects on rodent behavior and has the potential to rescue cognitive deficits^26–28^. In particular, we recently reported that ELFEF exposure enhances hippocampus-dependent learning and memory in mice^9,10^. By using a similar stimulation protocol Tasset and collegues^29^ found that ELFEF stimulation counteracted depressive-like behavior in a rat model of Huntington’s disease. Nonetheless, negative effects caused by ELFEF on memory were also reported in few studies^30,31^, suggesting that different experimental models may have varying effects, especially on the different exposure paradigms used. Thus, further research is necessary to elucidate ELFEF molecular mechanisms and its therapeutic potential.

Many factors including odor enrichment, context-dependent training, aging, and neurodegenerative diseases differentially modulate SVZ neurogenesis^32,33^. However, though extensive research focused on the cellular and developmental characterization of OB neurons, the behavioral changes accompanying this phenomenon are still not fully understood^11,12,19,34^. Previous studies demonstrated that neurogenesis is important for olfactory behavior^35–39^. In particular, animals showing reduced neurogenesis exhibited alterations in fine olfactory discrimination and short-term memory^34,40^, while mice exposed to an enriched environment presented both an increased neurogenesis and short-term olfactory memory^41^. Moreover mitral cell synchronization was found to be implicated in the odor information processing^42–44^. However, recent reports also suggested that genetic or pharmacological ablation of bulbar neurogenesis does not affect fine discrimination of odors and long-term olfactory memory performances^34,45^. Why different manipulations of adult neurogenesis lead to different behavioral outcomes is at present unknown. It is noteworthy, however, that behavioral paradigms used in these studies are different and OB neurogenesis might be involved in some but not all olfactory behaviors. In this context, our work links for the first time ELFEF stimulation to SVZ neurogenesis and changes in olfactory behavior.

Notably, we found that ELFEF exposure increased the number of NSCs proliferating in the SVZ, the progression of progenitor cells toward the differentiation and their migration in the RMS and OB, thus suggesting that ELFEF positively affects most steps of adult neurogenesis. Accordingly, we found that ELFEF activates the Wnt-β-catenin pathway that is critically involved in orchestrating neurogenesis^15,16^. In the Wnt/ β-catenin canonic signaling, Wnt binding to the Frizzled/LRP heteromeric receptors expressed in adult progenitor cells allows the downstream formation of the β-catenin TCF/LEF complex which leads to transcriptional activation of neurogenic genes and consequent regulation of adult neurogenesis. The presence of a large number of both Wnt and Frizzled members increases the complexity of this signaling. For example, activity-dependent changes in Wnt expression have been found following LTP induction in a mouse hippocampal slice model and a rapid regulation of Wnt proteins has been shown during memory consolidation in mice^46,47^. Interestingly, Wnt signaling was found to be necessary to consolidate different types of memory in mice^46,47^, and Wnt signaling impairment has been associated to memory deficits in mice and humans^48–50^. A recent study has indeed demonstrated that Wnt/β-catenin signaling plays a pivotal role in olfactory memory in Drosophila^25^, showing that increased Wnt expression leads to the accumulation of β-catenin in the mushroom bodies neurons which, in turn, orchestrates transcriptional modifications required for long-term memory storage^25^. This report is consistent with our finding that Wnt3 up-regulation after ELFEF exposure is followed by β-catenin accumulation in the nuclei of differentiating NSCs and paralleled by improved olfactory memory in mice. Notably, the blockade of Wnt/β-catenin pathway also reduced NSC proliferation and olfactory behavior in sham-exposed mice, thus supporting its physiological role in both functions.

Increased Wnt3 expression in the SVZ of ELFEF-exposed mice correlated with enhanced expression of the neurogenic genes Hes1 and Mash1. The role of these genes in neurogenesis has been studied extensively in both neurogenic niches. Hes1 is a downstream target of Notch signaling, which is essential for proliferation and maintenance of NSCs^20^ and Mash1 is a transcription factor involved in the production and commitment of neural precursor cells^22^. Notably, we did not find changes in other neurogenic transcriptional regulators of SVZ neurogenesis, such as Tlx, involved in neural stem cell self-renewal^21^, Pax6 and Prox1, implicated in fate determination^23,24^. Moreover, RT-qPCR analyses performed on hippocampal tissue extracts showed that the ELFEF-induced upregulation of Wnt3 expression was restricted to SVZ and it was not detectable in the hippocampus, although in the latter ELFEF regulated the expression of other neurogenic genes (e.g., Hes1 and Mash1), in agreement with our previous reports^9^. These findings suggest that ELFEF effects on SVZ rely on the activation of a specific pattern of genes and that the two neurogenic niches respond to ELFEF stimulation activating a non-completely overlapping gene signature. This is not surprising considering that the neurogenicity of SVZ and hippocampal NSCs is finely tuned by local signals, as already demonstrated by transplantation experiments showing that NSCs may loss or acquire different neurogenic potential if placed in non-spontaneously neurogenic regions or competent neurogenic niches respectively^51^.

*In vitro* blockade of Wnt signalling by Dkk-1 prevented the ELFEF-induced upregulation of Wnt3, Hes1 and Mash1 mRNAs. Likewise, *in vivo* Wnt signaling inhibition strongly impaired olfactory memory. These data further support our contention that Wnt signaling plays a critical role in mediating ELFEF effects on neurogenesis and olfactory memory.

A better understanding of the molecular mechanisms underlying ELFEF’s effects and optimization of the ELFEF stimulation protocol will be needed for translating these results to humans. The duration of ELFEF’s effects is also yet unknown and needs to be investigated along with the relative contribution of SVZ vs. hippocampal neurogenesis to olfactory memory, and their modulation by ELFEF, matters that are out of the scopes of the present study. Nonetheless data reported here characterize a new molecular mechanism relying on the Wnt/β-catenin pathway that regulates olfactory memory in mice and is selectively boosted by ELFEF in the SVZ. Hopefully, our findings may be exploited in the near future for the treatment of brain disorders associated with impaired adult neurogenesis and memory loss including aging and stress-related disorders.

## Methods

### Mice

Adult male C57BL/6 mice (Charles River Laboratories, Lecco, Italy) of 4–5 weeks old (average weight: ~20 g) were randomly divided into two groups: (1) sham-exposed mice (control) and (2) ELFEF-exposed mice. Mice were housed under a 12-h light-dark cycle at room temperature (RT) of 19–22 °C. Water and food were available *ad libitum* except for the cohorts that were tested in the long-term memory test. Efforts were made to limit the number of mice used and to minimize their pain and suffering. All experiments were approved by the Ethics Committee of the Catholic University and were in line with Italian (Ministry of Health guidelines, Legislative Decree No. 116/1992) and European Union (Directive No. 86/609/EEC) legislations on animal procedures.

ELFEF stimulation (1 mT; 50 Hz) was delivered by a solenoid surrounding a Plexiglas cylinder (diameter 20 cm; length 42 cm) and supplied by an AC power generator, as previously described^8,9^. For *in vivo* exposures, plastic housing cages containing mice were placed inside the solenoid^8,9^. For control mice, plastic housing cages containing mice were placed inside the solenoid, but the power generator was switched off. For *in vitro* experiments, the solenoid was placed in the CO_2_ incubator where NSCs isolated from the SVZ were cultured. Amplitude and frequency of ELFEF stimulation were continuously monitored as well as temperature of culture media to exclude any non-specific thermal effects, as previously described^9,10^.

For behavioral and immunofluorescence studies, mice were exposed 3.5 h/day for 12 days (12D × 3.5 h) (Supplementary Fig. S8a,b). For gene expression studies, western blot and immunofluorescence of β-catenin, mice were exposed to shorter ELFEF stimulation protocols (3.5 h/day for 4 days (4D × 3.5 h), Supplementary Fig. S8c) according to our previous studies^9^. For *in vitro* studies, NSCs were exposed to ELFEF for 3 h, 6 h and 24 h (Supplementary Fig. S8d,e).

### Behavioral Studies

#### Odor detection threshold test

The protocol for the threshold detection test was adapted from Perez-Villalba and colleagues^52^. Briefly, mice were placed in a rectangular plastic-covered wooden box (22 × 22 × 28 cm), with wooden bedding covering the floor. A 1-cm wide hole was made on a side of the box, positioned 8 cm from the ground floor and 10.5 cm from lateral walls. After 3 min of habituation to the box, a cotton-stick soaked in mineral oil (20 µL) was inserted in the cage hole and left in place for 1 min. This procedure was repeated 5 times, with an inter-trial interval (ITI) of 1 min, during which no cotton-stick was presented. After the 5^th^ ITI, mice were exposed to a cotton-stick soaked with the diluted odorant (Octanol, Sigma-Aldrich, Cat #W28001 or (S)-Limonene, Sigma-Aldrich, Cat. #W504505) at the lowest concentration (10^−7^%) for 1 min. The procedure was repeated until all the concentrations, following increasing order, were presented. The following concentrations were used: 10^−7^, 10^−5^, and 10^−4^%.

#### Innate preference test

The protocol for the innate odor-driven response was modified from Root and colleagues^18^. A plastic box with covered walls was used as behavioral apparatus. The floor was covered with wooden bedding. Mice were allowed to freely explore the apparatus for 10 min, after which a scented piece of paper (2 × 2 cm) was positioned in one corner of the cage. In the next 15 minutes the animal behavior was recorded and the time spent in each quadrant was calculated using EthoVision XT video tracking system (Wageningen, The Netherlands). The behavioral response to an array of odorants (attraction *vs*. repulsion) was quantified by a performance index that represents the percentage difference from chance occupancy in the lower right quadrant, where the odor was placed (performance index = (P − 25)/0.25, where P is the percentage time in the odor containing quadrant). Couples of odors with different emotional values were used: cat urine (20 µL), rat urine (20 µL), Isoamyl acetate (10^−3^ M, Sigma-Aldrich, Cat. #W205532), S-Limonene (0.2%), mineral oil (Sigma-Aldrich, Cat. #M3516), peanut butter (10%, 40 µL), and female mouse urine (20 µL).

#### Odor discrimination

The olfactory cross-habituation task assesses the ability of mice to discriminate between odorants by habituating them to an odorant (Hab) and measuring their cross-habituation to a second odorant (Dishab). If the second odorant is not discriminated from the first, it does not elicit an increased investigation response by the mouse. Odors were presented by placing 60 µL of the odor onto a filter paper disc (Whatman No. 1, Sigma-Aldrich, St. Louis, MO) contained within a pipette that was placed on top of the wire cage lid. The test session consisted of four 50-s presentations of the habituation odor, (R)-Limonene (0.2% v/v, Sigma-Aldrich, Cat. #W263303) or (+)-Carvone (0.2% v/v, Sigma-Aldrich, Cat. #22070), at 5-min intervals, in which the mice are placed in their home cages, followed by one 50-s presentation of the test odorant, (S)-Limonene (0.2% v/v) or (−)-Carvone (0.2% v/v, Cat. #22060). The time spent sniffing each presented odor within 1 cm of the odor source was measured (investigation time). Odors were renewed between each test. Only mice that investigated an odor for at least 1 s during its first presentation were included in the analysis. The odorant pair was chosen based upon its perceptual similarity and its activation of overlapping regions of the glomerular cell layer^53^.

#### Olfactory short-term memory

The olfactory short-term memory test evaluates a mouse’s ability to recognize a familiar odor. Mice were exposed to the same odor (Isoamyl acetate 10^−3^ M or Octanol 1 mM) during two sessions of 5 min each that were separated by a 60-min interval. The pipette containing 60 µL of the odor onto a filter paper disc (Whatman No. 1) was inserted randomly in the cage grid to avoid a spatial recognition effect. A decrease in the investigation time (mean duration) on the second odor exposure was considered as an indication that the mouse remembered the odor^34^.

#### Long-term odor-associative memory

The long-term olfactory memory test was used in order to study the ability to learn and memorize the long-term association between an odor and a reward (food). All mice were weighed daily and fed a standard diet (RIEPER, Vandoies, Italy) to maintain 85% of their free feeding weight. Water was available *ad libitum*. After 1 week, mice were subjected to 4 training sessions per day for 4 days. At each session, mice were placed into an arena (21 × 25 × 46 cm), with only 1 of the 2 odors (with or without the food) for 10 min, then they were brought back in their home cage for another 10 min (ITI) before the next training.

Twenty-four hours after training, the test trial was administered. The 2 odors were presented simultaneously in the same apparatus without any kind of reward. The time a mouse spent digging the bedding in each cup containing the odors (all at a concentration of 10^−3^ M) was recorded during a 2-min period and a preference index, calculated as digging time near the odor previously rewarded/total digging time, was measured. The following odors were used: (+)-Carvone: (6.4 M), Linalool (1 mM, Sigma-Aldrich, Cat. #L2602) were presented in association with a sugar reward and (−)-Carvone (6.4 M) and Phenyl acetate (1 mM, Sigma-Aldrich, Cat. # 108723) were presented without sugar^34^.

### Intra-ventricular injections

Mice were anesthetized with ketamine (100 mg/kg, Ketavet 100, MSD Animal Health S.r.l., Italy) and medetomidine (1 mg/kg, Dorbene vet, Zoetis S.r.l., Italy) and placed in a stereotaxic apparatus (David Kopf Instruments, USA) with a mouse adapter and lateral ear bars. The head skin was cut longitudinally and guide cannulae directed at the lateral ventricles (7 mm in length, 0.5 mm in diameter, Unimed, Cat. # tube AISI 316 L) were fixed on the calvarium with acrylic cement (3 M, Durelon, Cat. # 38019). The following coordinates with lambda and bregma in the same horizontal plane were used: anterior to bregma 0.46 mm; lateral to midline 0.8 mm; ventral from the dura 1.8 mm. Mice were monitored daily with appropriate post-surgical care for at least 1 week following surgery^54^.

Following a 1-week recovery period, mice were exposed to ELFEF for 4 days (4D × 3.5 h) for molecular analysis or 12 days (12D × 3.5 h) for behavioral experiments. Animals received intra-ventricular vehicle (phosphate buffered saline (PBS), 0.5 μL) or Dickkopf-1 (Dkk-1, R&D Systems; Cat. #5439-DK-010, Lot: SMR3415021) (200 ng/0.5 μL) infusions at a rate of 0.1 μL/min, every day, before ELFEF exposure. For RT-qPCR and western blot experiments, mice were sacrificed the day after last ELFEF exposure. For immunofluorescence experiments, mice were exposed for 4 or 12 days and sacrificed the day after last ELFEF exposure. For behavioral experiments, mice were administered olfactory tests one month after the end of the ELFEF stimulation protocol (D42).

### Neural Stem Cell cultures

Postnatal SVZ NSC culture were isolated according to previously published protocols^55^. Briefly, brains of newborn (0–1 day old) C57BL/6 mice were microdissected to obtain the lateral ventricle region upon sagittal sectioning. Tissues were finely minced and digested by accutase (in DPBS, 0.5 mM EDTA; Innovative Cell Technologies, Inc., San Diego, CA, USA) at 37 °C for 30 min. After centrifugation, cells were carefully dissociated by passaging in fire-polished Pasteur pipettes and resuspended in NeurobasalA medium, supplemented with 2% B27 (Gibco, Grand Island, NY, USA), Glutamax (0.5 mM; Invitrogen, Carlsbad, CA), mouse fibroblast growth factor 2 (FGF2, 10 ng/mL; Invitrogen), epidermal growth factor (EGF, 10 ng/mL; Invitrogen), mouse platelet-derived growth factor bb (PDGFbb, 10 ng/mL; Invitrogen).

Cells were seeded onto 25-cm^2^ T-flask and incubated at 37 °C in 5% CO_2_ atmosphere. During the first week of *in vitro* culture NSCs began to form neurospheres. At 2-day intervals, the neurospheres were collected and passaged by a gently enzymatic and mechanical dissociation.

To obtain monolayer cultures, neurospheres of established cultures were passaged by enzymatic and mechanical dissociation and plated as single cells onto Matrigel Matrix (Becton Dickinson, Franklin Lakes, NJ) pre-coated Petri dishes. NSCs cultured in the medium described above remained in an undifferentiated state and proliferated. To induce differentiation, NSCs were cultured for 3, 6 or 24 hours in differentiation medium NeuroCult basal medium complemented with NeuroCult differentiation supplement (StemCell technologies, Inc., Vancouver, Canada). *In vitro* treatments with Wnt signaling inhibitor were performed in SVZ NSC cultured for 24 h in the presence of 100 ng/mL Dkk-1.

### Tissue isolation and collection for RT-qPCR and Western blotting analyses

As described above, the day after the last ELFEF stimulation, sham or ELFEF-exposed mice, with or without Dkk-1 injection, were anesthetized by saturated isoflurane vapor and quickly decapitated. Brains were removed under aseptic conditions and placed into Petri dishes containing ice-cold sterilized Dulbecco’s Phosphate-Buffered Saline pH 7.4 (1×) (DPBS, Invitrogen, Long Island, NY). Coronal brain sections containing SVZ and DG of hippocampus were cut and microdissected under a high-magnification microscope with microscalpels, as previously reported by Guo and collegues^56^ with minor modifications. The lateral ventricle area or hippocampus in each slice of the same animal was pooled, rapidly frozen in liquid nitrogen and stored at −80 °C.

### RT-qPCR experiments

All reverse transcription-quantitative PCR (RT-qPCR) experiments were carried out as previously reported^9^. The donor mouse was anesthetized with a cocktail of ketamine (100 mg/kg) and medetomidine (1 mg/kg) and then sacrificed. The expression of neurogenic genes hairy and enhancer of split-1 (Hes1), Achaete-scute complex homolog-1 (Mash1) and Wingless-Type MMTV Integration Site Family, Member 3 (Wnt3), Paired Box 6 (Pax6), Prospero Homeobox 1 (Prox1), T-Cell Leukemia Homeobox 1 (Tlx1) were analyzed in both SVZ tissues from control and ELFEF-exposed mice, and in SVZ NSCs of newborn mice exposed *in vitro* to control and ELFEF stimulation.

Briefly, total RNA was extracted using an RNAqueous Micro Kit (Ambion Inc., Austin, TX, USA) according to the manufacturer’s instructions. Equal amounts of RNA (2 μg) were subsequently reverse-transcribed using a high-capacity cDNA reverse transcription kit (Applied Biosystems, Foster City, CA, USA). Quantitative RT-PCR was performed in triplicates using inventoried TaqMan Gene expression assays purchased from Applied Biosystems. Relative mRNA levels for genes of interest were normalized to TATA-box-binding protein (TBP), taken as housekeeping gene, and calculated by using the 2^−ΔΔCt^ method^57^. Three independent experiments were performed with the ABI 7500 Sequence Detection System Analyzer for RT-qPCR (Applied Biosystems). Results were expressed as mean fold changes induced by ELFEF exposure compared with control samples.

### BrdU injection and *ex vivo* immunofluorescence assays

To determine the effects of ELFEF on NSC proliferation control and ELFEF-exposed mice received a single intraperitoneal (i.p.) injection of 5-bromo-2′-deoxyuridine (BrdU) (150 mg/kg dissolved in sterile 0.9% NaCl solution; Sigma, Milan, Italy, Cat. #B9285) and were sacrificed 2 hours later. To assess the effects of ELFEF on mature neurons, control and ELFEF-treated mice were injected i.p. with BrdU (100 mg/kg) for the first 5 days of exposure and sacrificed 30 days after the final stimulation session^9^. At the end of treatments mice were deeply anesthetized with ketamine (100 mg/kg) and medetomidine (1 mg/kg); they were transcardially perfused with an oxygenated Ringer’s solution (pH 7.3), followed by 4% paraformaldehyde (PFA) in 0.1 M PBS (pH 7.4) and brains were collected, post-fixed overnight at 4 °C and then transferred to a solution of 30% sucrose in 0.1 M PBS. Brains were sectioned sagittally (20-μm thick) to better visualize the migration of SVZ neuroblasts to the OB, or coronally (40-μm thick)^6,11,58,59^ by a cryostat (SLEE, Mainz, Germany) and sections were stored at −20 °C until use.

Immunolabeling of BrdU, Nestin, doublecortin (DCX), NeuN, Ki67 and β-Catenin was performed as previously described^9,10,60^ with slight modifications. Rat monoclonal anti-BrdU antibody (1:400, Abcam, Cambridge, UK, Cat. #ab6326), rabbit anti-DCX antibody (1:250, Cell Signaling Cat. #4604), mouse monoclonal anti-NeuN and mouse monoclonal anti-Nestin antibodies (1:150, Millipore Cat. #MAB377 and #MAB353, respectively), rabbit polyclonal anti Ki67 and rabbit anti-β-Catenin (1:200, Abcam, Cat. #ab15580 and #ab6302, respectively) were used as primary antibodies. Secondary antibodies Alexa Fluor-488 donkey anti-rat IgG (1:500, Cat. #A21208), Alexa Fluor-546 donkey anti-rabbit IgG (1:500, Cat. #A10040), and Alexa Fluor-546 donkey anti-mouse (1:500, Cat. #A10036) were purchased from Invitrogen.

Confocal images were obtained with a Nikon A1 MP system connected to an Eclipse T-i microscope equipped with a 20×, 40× and 60× objectives. BrdU/Nestin- and Ki67-positive cells were counted in all the SVZ by acquiring 15-µm thick Z-stacks (one XY plane every 0.5 µm) of 20× images at a resolution of 1024 × 1024 pixels or 40× (with further 2× magnification) at 512 × 512 pixels. BrdU^+^/DCX^+^ cells were counted in the RMS by acquiring 15-µm thick Z-stacks of 40× images at a resolution of 512 × 512 pixels. At least three sections were analyzed for each brain. BrdU^+^/NeuN^+^ cells were counted by acquiring 15-µm thick Z-stacks of 40× images at a resolution of 512 × 512 pixels. Four sectors (two rostrals and two caudals) were analyzed for each slice. β-Catenin immunofluorescence was acquired at 63× and 512 × 512 pixels. Nuclei were counterstained with DAPI.

### Immunocytochemistry

NSCs were fixed in 4% PFA for 10 min and permeabilized in 1 × PBS/Triton X-100 (0.3%) for 15 min. Non-specific binding sites were blocked by incubation in 10% BSA for 20 min and thereafter, primary antibody (anti-β-Catenin, 1:1000, polyclonal, Abcam, Cat. #ab6302) was added to the cells. The following day, after a brief wash, cells were incubated with the FITC-conjugated secondary antibody (anti-rabbit, 1:200, Jackson Laboratory) for 1 h at RT; nuclei were counterstained with DAPI (0.5 mg/mL; Invitrogen) for 10 min. Images were obtained with a Nikon Eclipse T-i confocal microscope. All experiments were repeated independently 3 times.

### Western Blotting

Cytoplasmic and nuclear protein fractions were isolated from NSCs by means of NE-PER Extraction Kit (Thermo Fisher Scientific). Total extracts from SVZ and hippocampal regions of control mice were lysed in RIPA buffer solution (Pierce, Rockford, IL, USA) containing 50 mM Tris, 150 mM NaCl, 1 mM EDTA, 1% sodium deoxycholate, 1% TX-100, 0.1% sodium dodecyl sulfate (SDS) and ×1protease inhibitor mixture (Sigma), 1 mM sodium orthovanadate, and 1 mM NaF. All samples were resolved by Western blot standard procedures^61–63^, using the following antibodies: polyclonal anti-β-Catenin antibody (1:4000, Abcam, Cat. #ab6302), monoclonal anti-actin antibody (1:4000, Sigma, Cat. #T6074), monoclonal anti-GAPDH antibody (1:2000, Abcam, Cat. #ab8245), monoclonal anti-fibrillarin (1:1000, Thermo Scientific, Cat. #MA3-16771). All experiments were repeated independently 3 times.

### Statistical analysis

Sample sizes choices were based on previous data sets and studies, including our own that used similar methods, to achieve reliable statistical power (0.8). Sample estimation and statistical analysis were performed using SigmaPlot 10.0, StatView v. 5.0 (SAS Institute) and GraphPad 5 softwares. Data were first tested for equal variance and normality (Shapiro-Wilk test or D’Agostino & Pearson normality test) and then subjected to the proper statistical tests (i.e., Student’s *t*-test, Mann-Whitney test, one-way ANOVA, two-way ANOVA, two-way RM ANOVA) as indicated in the main text for each experiment. Post-hoc multiple comparisons were performed with Bonferroni correction. All statistical tests were two-tailed and the level of significance was set at 0.05. Results were presented as means ± SEM. Criteria of mouse exclusion/inclusion were pre-established according to Ethics Committee guidelines but no data were excluded from analysis. All experimenters were blind to treatment.

### Data Availability

All data generated or analyzed during this study are included in this published article (and its Supplementary Information files).

## Electronic supplementary material

Supplementary Information

## References

[1] Abrous DN, Koehl M, Le Moal M (2005). Adult neurogenesis: from precursors to network and physiology. Physiol. Rev..

[2] Gage FH (2000). Mammalian neural stem cells. Science.

[3] Kelsch W, Sim S, Lois C (2010). Watching synaptogenesis in the adult brain. Annu. Rev. Neurosci..

[4] Brown J (2003). Enriched environment and physical activity stimulate hippocampal but not olfactory bulb neurogenesis. Eur. J. Neurosci..

[5] Czéh B (2002). Chronic psychosocial stress and concomitant repetitive transcranial magnetic stimulation: effects on stress hormone levels and adult hippocampal neurogenesis. Biol. Psychiat..

[6] Arias-Carrión O (2004). Neurogenesis in the subventricular zone following transcranial magnetic field stimulation and nigrostriatal lesions. J. Neurosci. Res..

[7] Piacentini R, Ripoli C, Mezzogori D, Azzena GB, Grassi C (2008). Extremely low-frequency electromagnetic fields promote *in vitro* neurogenesis via upregulation of Ca(v)1-channel activity. J. Cell. Physiol..

[8] Cuccurazzu B (2010). Exposure to extremely low-frequency (50 Hz) electromagnetic fields enhances adult hippocampal neurogenesis in C57BL/6 mice. Exp. Neurol..

[9] Leone L (2014). Epigenetic Modulation of Adult Hippocampal Neurogenesis by Extremely Low-Frequency Electromagnetic Fields. Mol. Neurobiol..

[10] Podda MV (2014). Extremely low-frequency electromagnetic fields enhance the survival of newborn neurons in the mouse hippocampus. Eur. J. Neurosci..

[11] Valley MT, Mullen TR, Schultz LC, Sagdullaev BT, Firestein S (2009). Ablation of mouse adult neurogenesis alters olfactory bulb structure and olfactory fear conditioning. Front. Neurosci..

[12] Lazarini F, Lledo P-M (2011). Is adult neurogenesis essential for olfaction?. Trends Neurosci..

[13] Varela-Nallar L, Inestrosa NC (2013). Wnt signaling in the regulation of adult hippocampal neurogenesis. Front. Cell. Neurosci..

[14] Kuwabara T (2009). Wnt-mediated activation of NeuroD1 and retro-elements during adult neurogenesis. Nat. Neurosci..

[15] Azim K (2014). Persistent Wnt/beta-catenin signaling determines dorsalization of the postnatal subventricular zone and neural stem cell specification into oligodendrocytes and glutamatergic neurons. Stem Cells.

[16] Wu MV, Hen R (2013). The Young and the Restless: Regulation of Adult Neurogenesis by Wnt Signaling. Cell Stem Cell.

[17] Doty RL, Kamath V (2013). The influences of age on olfaction: a review. Front. Psychol..

[18] Root CM, Denny CA, Hen R, Axel R (2014). The participation of cortical amygdala in innate, odour-driven behaviour. Nature.

[19] Alonso M (2012). Activation of adult-born neurons facilitates learning and memory. Nat. Neurosci..

[20] Bai G (2007). Id sustains Hes1 expression to inhibit precocious neurogenesis by releasing negative autoregulation of Hes1. Dev. Cell.

[21] Liu HK (2008). The nuclear receptor tailless is required for neurogenesis in the adult subventricular zone. Genes Dev..

[22] Kim EJ, Ables JL, Dickel LK, Eisch AJ, Johnson JE (2011). Ascl1 (Mash1) defines cells with long-term neurogenic potential in subgranular and subventricular zones in adult mouse brain. PLoS ONE.

[23] Ninkovic J (2010). The transcription factor Pax6 regulates survival of dopaminergic olfactory bulb neurons via crystallin alpha. Neuron.

[24] Bunk EC (2016). Prox1 Is Required for Oligodendrocyte Cell Identity in Adult Neural Stem Cells of the Subventricular Zone. Stem Cells.

[25] Tan Y, Yu D, Busto GU, Wilson C, Davis RL (2013). Wnt signaling is required for long-term memory formation. Cell Rep..

[26] Liu T, Wang S, He L, Ye K (2008). Chronic exposure to low-intensity magnetic field improves acquisition and maintenance of memory. Neuroreport.

[27] Wassermann EM, Lisanby SH (2001). Therapeutic application of repetitive transcranial magnetic stimulation: a review. Clin. Neurophysiol..

[28] Medina FJ, Tunez I (2010). Huntington’s disease: the value of transcranial meganetic stimulation. Curr. Med. Chem..

[29] Tasset I (2012). Neuroprotective effects of extremely low-frequency electromagnetic fields on a Huntington’s disease rat model: effects on neurotrophic factors and neuronal density. Neuroscience.

[30] Mostafa RM, Mostafa YM, Ennaceur A (2002). Effects of exposure to extremely low-frequency magnetic field of 2 G intensity on memory and corticosterone level in rats. Physiol. Behavior.

[31] Zhou H, Chen G, Chen C, Yu Y, Xu Z (2012). Association between extremely low-frequency electromagnetic fields occupations and amyotrophic lateral sclerosis: a meta-analysis. PLoS One.

[32] Ma DK, Kim WR, Ming GL, Song H (2009). Activity-dependent extrinsic regulation of adult olfactory bulb and hippocampal neurogenesis. Ann. NY Acad. Sci..

[33] Sultan S (2010). Learning-dependent neurogenesis in the olfactory bulb determines long-term olfactory memory. FASEB J..

[34] Breton-Provencher V, Lemasson M, Peralta MR, Saghatelyan A (2009). Interneurons produced in adulthood are required for the normal functioning of the olfactory bulb network and for the execution of selected olfactory behaviors. J. Neurosci..

[35] Sakamoto M (2011). Continuous neurogenesis in the adult forebrain is required for innate olfactory responses. Proc. Natl. Acad. Sci. USA.

[36] Garrett L (2015). Conditional reduction of adult born doublecortin-positive neurons reversibly impairs selective behaviors. Front. Behav. Neurosci..

[37] Moreno MM (2009). Olfactory perceptual learning requires adult neurogenesis. Proc. Natl. Acad. Sci. USA.

[38] Lazarini F (2009). Cellular and behavioral effects of cranial irradiation of the subventricular zone in adult mice. PLoS One.

[39] Arruda-Carvalho M (2014). Posttraining ablation of adult-generated olfactory granule cells degrades odor-reward memories. J. Neurosci..

[40] Enwere E (2004). Aging results in reduced epidermal growth factor receptor signaling, diminished olfactory neurogenesis, and deficits in fine olfactory discrimination. J. Neurosci..

[41] Rochefort C, Gheusi G, Vincent JD, Lledo PM (2002). Enriched odor exposure increases the number of newborn neurons in the adult olfactory bulb and improves odor memory. J. Neurosci..

[42] Laurent G (2001). Odor encoding as an active, dynamical process: experiments, computation, and theory. Annu. Rev. Neurosci..

[43] Saghatelyan A (2005). Activity-dependent adjustments of the inhibitory network in the olfactory bulb following early postnatal deprivation. Neuron..

[44] Lledo PM, Lagier S (2006). Adjusting neurophysiological computations in the adult olfactory bulb. Semin. Cell Dev. Biol..

[45] Imayoshi I (2008). Roles of continuous neurogenesis in the structural and functional integrity of the adult forebrain. Nat. Neurosci..

[46] Maguschak KA, Ressler KJ (2011). Wnt signaling in amygdala-dependent learning and memory. J. Neurosci..

[47] Chen J, Park CS, Tang SJ (2006). Activity-dependent synaptic Wnt release regulates hippocampal long term potentiation. J. Biol. Chem..

[48] Seib D (2013). Loss of Dickkopf-1 restores neurogenesis in old age and counters cognitive decline. Cell Stem Cell.

[49] De Ferrari GV, Inestrosa NC (2000). Wnt signaling function in Alzheimer’s disease. Brain Res. Rev..

[50] Caricasole A (2004). Induction of Dickkopf-1, a negative modulator of the Wnt pathway, is associated with neuronal degeneration in Alzheimer’s brain. J. Neurosci..

[51] Alvarez-Buylla A, Lim DA (2004). For the long run: maintaining germinal niches in the adult brain. Neuron..

[52] Perez-Villalba A, Palop MJ, Perezz-Sánchez F, Fariñas I (2015). Assessment of Olfactory Behavior in Mice: Odorant Detection and Habituation-Dishabituation Tests. Bio-protocol.

[53] Mandairon N, Stack C, Kiselycznyk C, Linster C (2006). Enrichment to odors improves olfactory discrimination in adult rats. Behav. Neurosci..

[54] Gaglio D (2014). Learning induced epigenetic modifications in the ventral striatum are necessary for long-term memory. Behav. Brain Res..

[55] Brewer GJ, Torricelli JR (2007). Isolation and culture of adult neurons and neurospheres. Nat. Protoc..

[56] Guo W, Patzlaff NE, Jobe EM, Zhao X (2012). Isolation of multipotent neural stem or progenitor cells from both the dentate gyrus and subventricular zone of a single adult mouse. Nat. Protoc..

[57] Livak KJ, Schmittgen TD (2001). Analysis of relative gene expression data using real-time quantitative PCR and the 2(-Delta Delta C(T)) *Method*. Methods.

[58] Langenfurth A (2016). Decreased demand for olfactory periglomerular cells impacts on neural precursor cell viability in the rostral migratory stream. Sci. Rep..

[59] Shinohara R (2012). A role for mDia, a Rho-regulated actin nucleator, in tangential migration of interneuron precursors. Nat. Neurosci..

[60] Fusco S (2016). A CREB-Sirt1-Hes1 Circuitry Mediates Neural Stem Cell Response to Glucose Availability. Cell Rep..

[61] Podda MV (2016). Anodal transcranial direct current stimulation boosts synaptic plasticity and memory in mice via epigenetic regulation of Bdnf expression. Sci. Rep..

[62] Podda MV (2012). Expression of olfactory-type cyclic nucleotide-gated channels in rat cortical astrocytes. Glia.

[63] Ripoli C (2014). Intracellular accumulation of amyloid-β (Aβ) protein plays a major role in Aβ-induced alterations of glutamatergic synaptic transmission and plasticity. J. Neurosci..

