# Supplementary material for: Olfactory memory is enhanced in mice exposed to extremely low-frequency electromagnetic fields via Wnt/β-catenin dependent modulation of subventricular zone neurogenesis
Source: Sci Rep. 2018 Jan 10;8:262. doi: 10.1038/s41598-017-18676-1 (PMC5762682; doi:10.1038/s41598-017-18676-1)
Supplement: Supplementary file 1 — Supplementary Information [file 41598_2017_18676_MOESM1_ESM.pdf]

Supplementary Information

Supplementary Figures 1-8

Olfactory memory is enhanced in mice exposed to extremely low-frequency electromagnetic fields via Wnt/β-catenin dependent modulation of subventricular zone neurogenesis

Alessia Mastrodonato, Saviana Antonella Barbati, Lucia Leone, Claudia Colussi, Katia Gironi, Marco Rinaudo, Roberto Piacentini, Christine Ann Denny, and Claudio Grassi

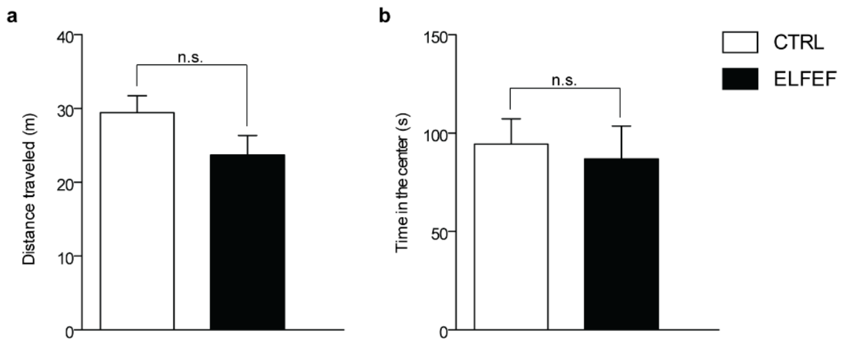

Supplementary Figure 1. ELFEF exposure does not affect locomotor activity. (***a***) Bar graphs showing the distance travelled by control (n = 8) and ELFEF-exposed mice (n = 8) (P=0.12, unpaired Student's *t*-test). (***b***) Bar graphs comparing the time spent in the center of the open field by control and ELFEF-exposed mice (P=0.72, unpaired Student's *t*-test). Data are expressed as means ± SEM. n.s.= not significant.

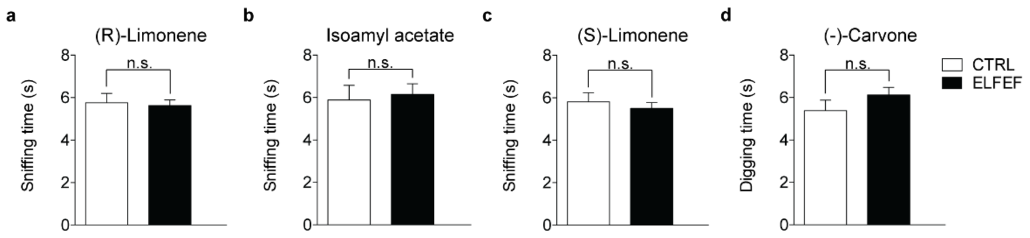

Supplementary Figure 2. Olfactory behavior was independent of specific odors. Bar graphs showing that in ELFEF-exposed mice (n = 8) the sniffing time of (R)-Limonene (***a***), Isoamyl acetate (***b***) and (S)-Limonene (***c***), and the digging time of (-)-Carvone (***d***) were not significantly different from controls (n = 8). Data are expressed as means ± SEM. n.s.= not significant.

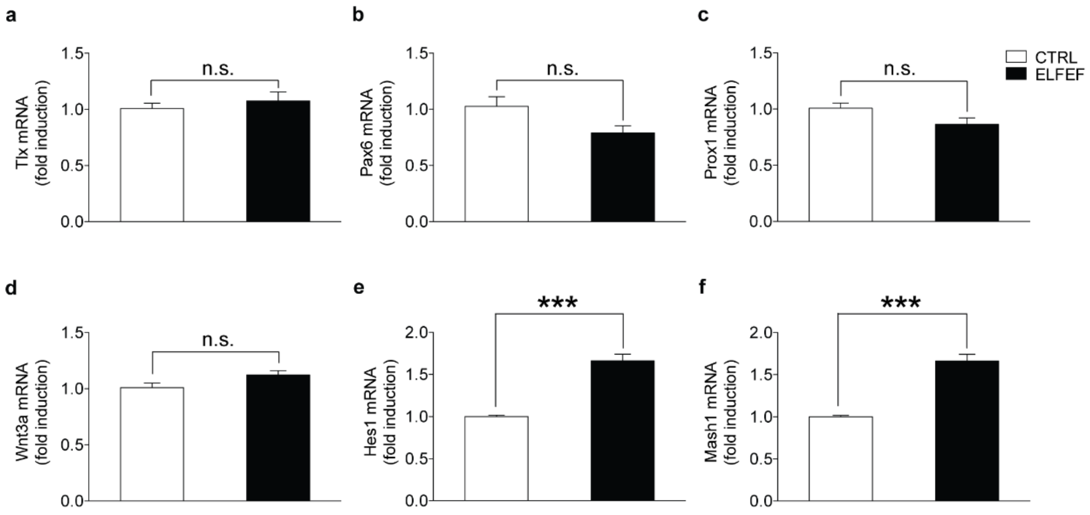

Supplementary Figure 3. Selectivity of ELFEF's effect on neurogenic gene expression. Upper panels showing RT-qPCR analysis of neurogenic genes unaffected by ELFEF stimulation in the SVZ: (***a***) Tlx, (***b***) Pax, and (***c***) Prox1 mRNA in SVZ extracts of control and ELFEF-exposed mice. Lower panels show mRNA levels of Wnt3a (***d***), Hes1 (***e***) and Mash1 (***f***) in hippocampal extracts of control and ELFEF mice. Notably, ELFEF significantly enhanced mRNA levels of Hes1 and Mash1 without affecting Wnt3a mRNA levels in the hippocampus. Values are expressed as means ± SEM of fold increase in the ratio of each gene/TBP, with the value of control mice taken as 1.0 (n = 4 mice per group, n.s.= not significant, *** p<0.001 unpaired Student's *t*-test).

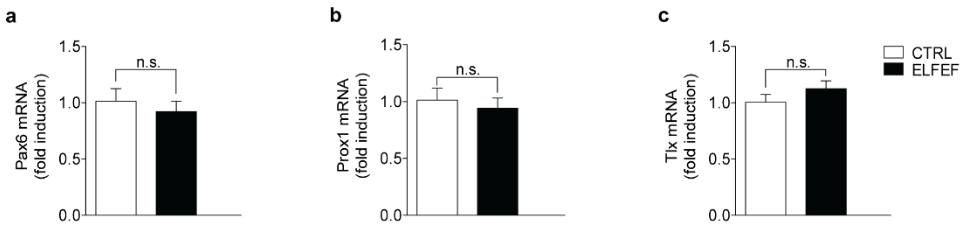

Supplementary Figure 4. ELFEF stimulation does not change the expression of some neurogenic genes in cultured NSCs isolated from the SVZ. Bar graphs (***a***-***c***) show unchanged levels of mRNA encoding for Pax6 (***a***), Prox1 (***b***), and Tlx (***c***) in ELFEF-exposed NSC extracts when compared to control extracts. Values are expressed as means ± SEM of the fold increase in the ratio of each gene/TBP, with the value of control (unexposed) NSCs taken as 1.0. Experiments were performed in triplicate. n.s.= not significant.

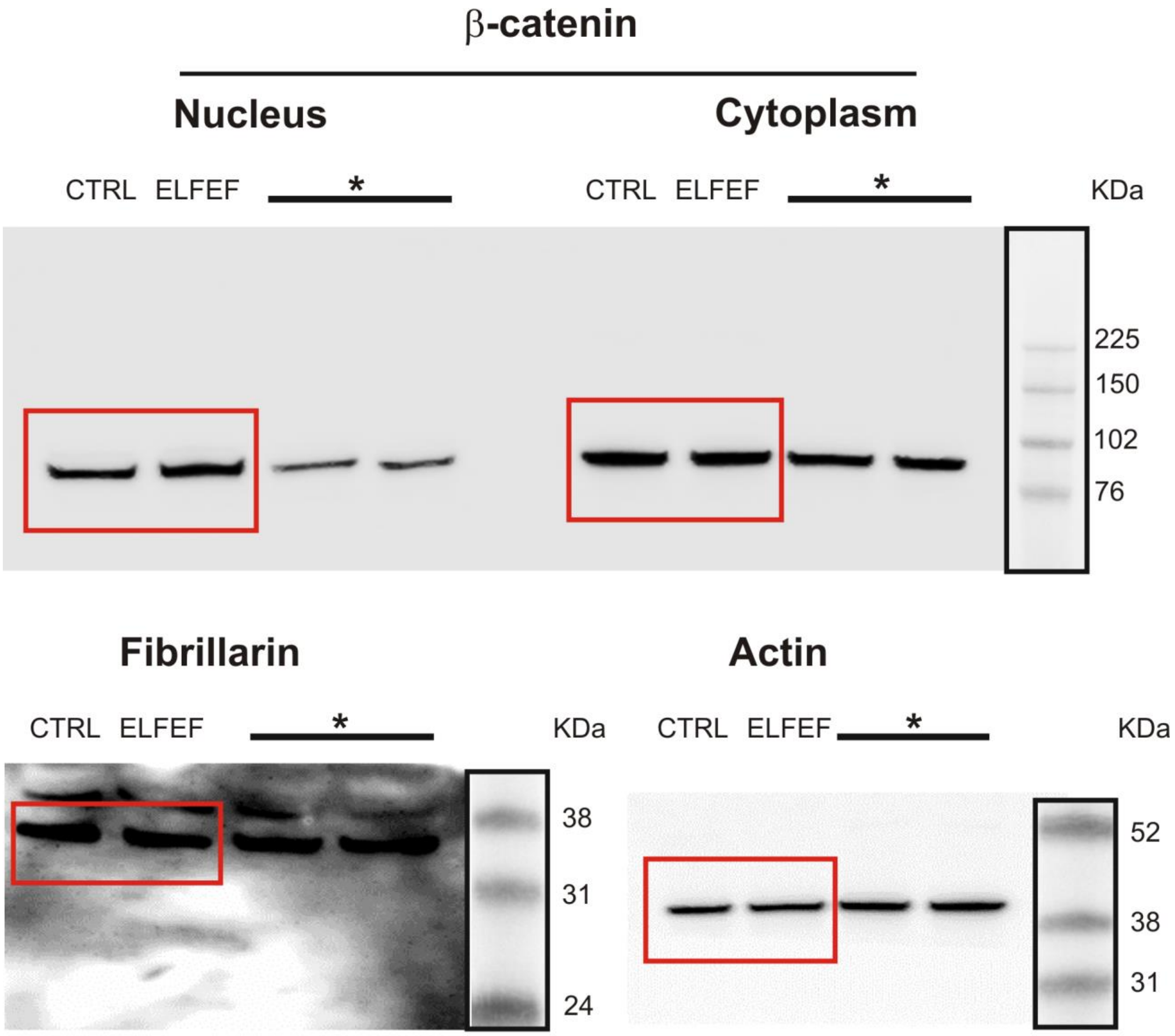

Supplementary Figure 5. Full length blots of data shown in Fig. 6*d*. Red boxes indicate bands reported in Fig. 6*d*. Lanes indicated with asterisks are loaded with samples that are not related to data presented in this manuscript. Molecular size markers are shown on the right side of each blot.

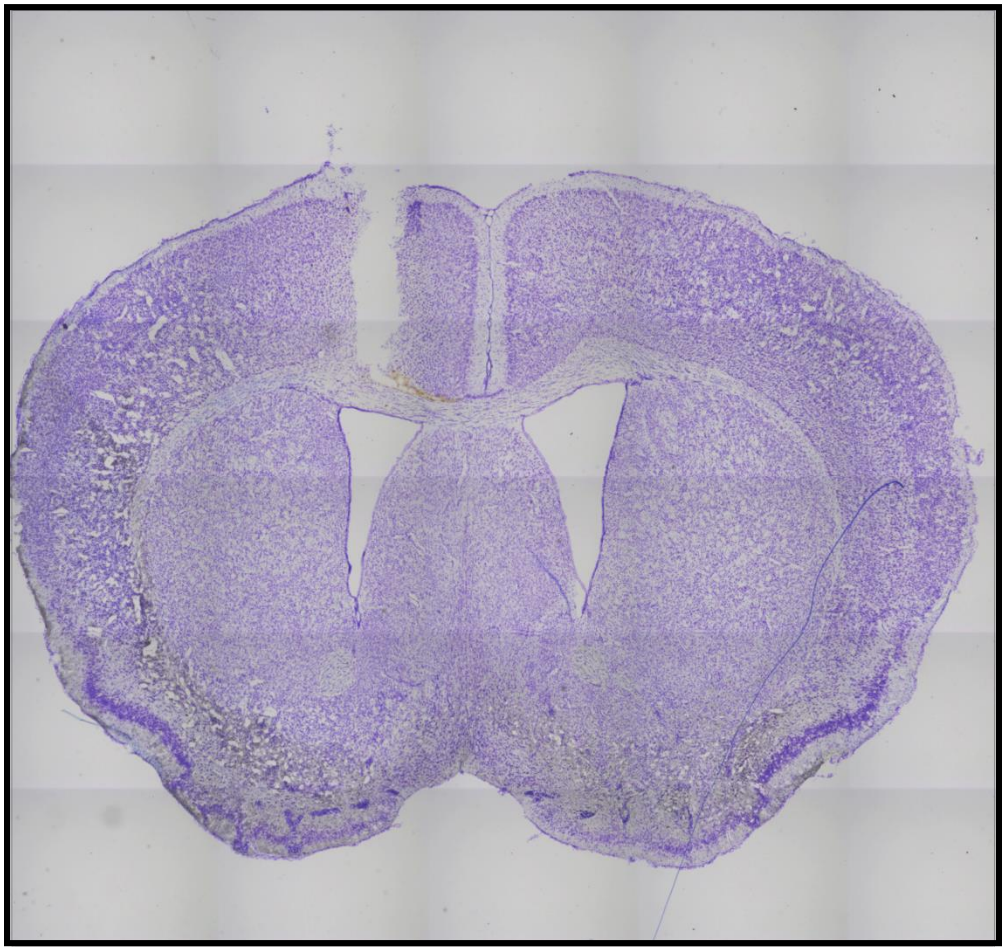

Supplementary Figure 6. Coronal brain section stained with Nissl showing the injection site of Dkk-1 into the lateral ventricle.

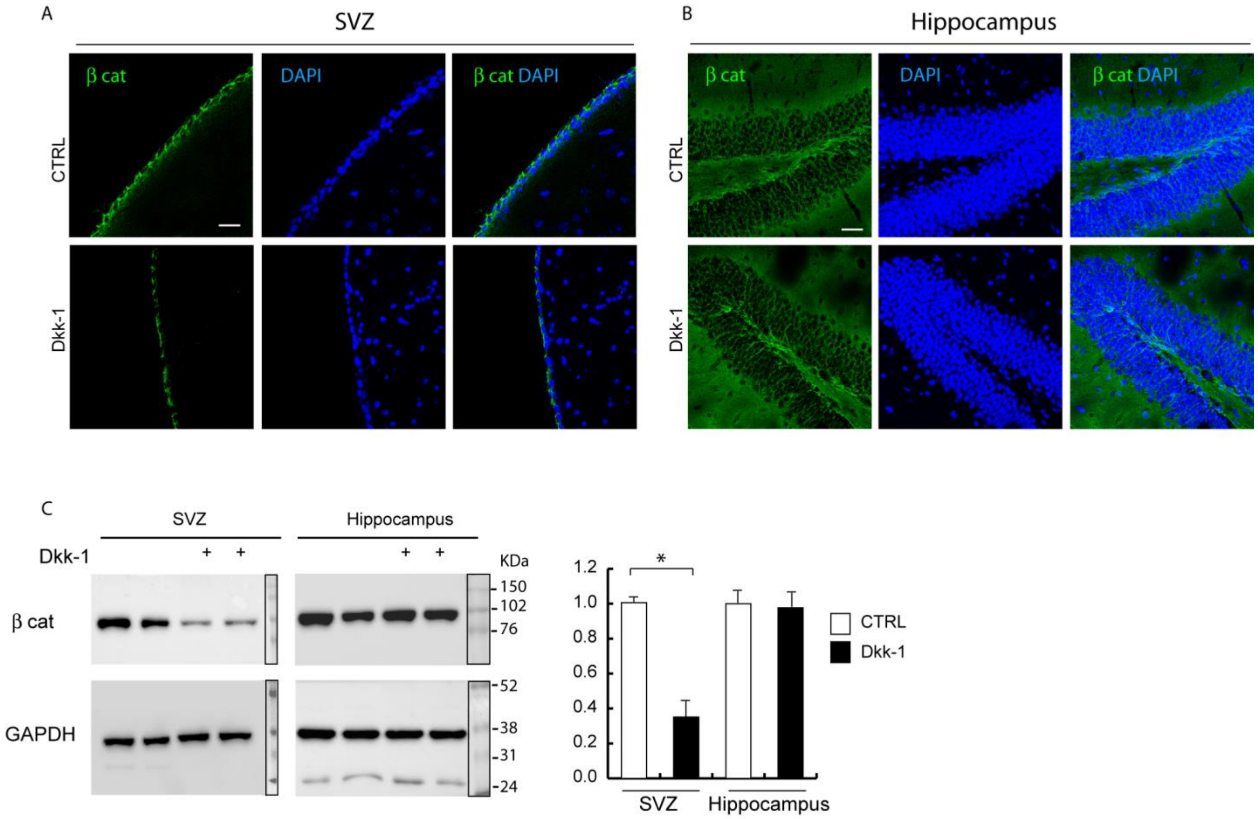

Supplementary Figure 7. Analysis of β-catenin expression in Dkk-1-injected mice. Confocal microscopy analysis in the SVZ (***a***) and in the hippocampus (***b***) from vehicle (CTRL) and Dkk-1-injected mice (Dkk-1) showing β-catenin immunoreactivity (green). Nuclei were counterstained with DAPI (blue) (scale bar: 25 μm, n = 4). Western blotting evaluation of β-catenin levels in SVZ and hippocampal tissue extracts from CTRL and Dkk-1 mice (***c***). The bar graph shows the densitometric analysis. GAPDH was used as loading control (n = 4 mice per group, *$p<0.05$ unpaired Student's *t*-test). Values are expressed as means ± SEM.

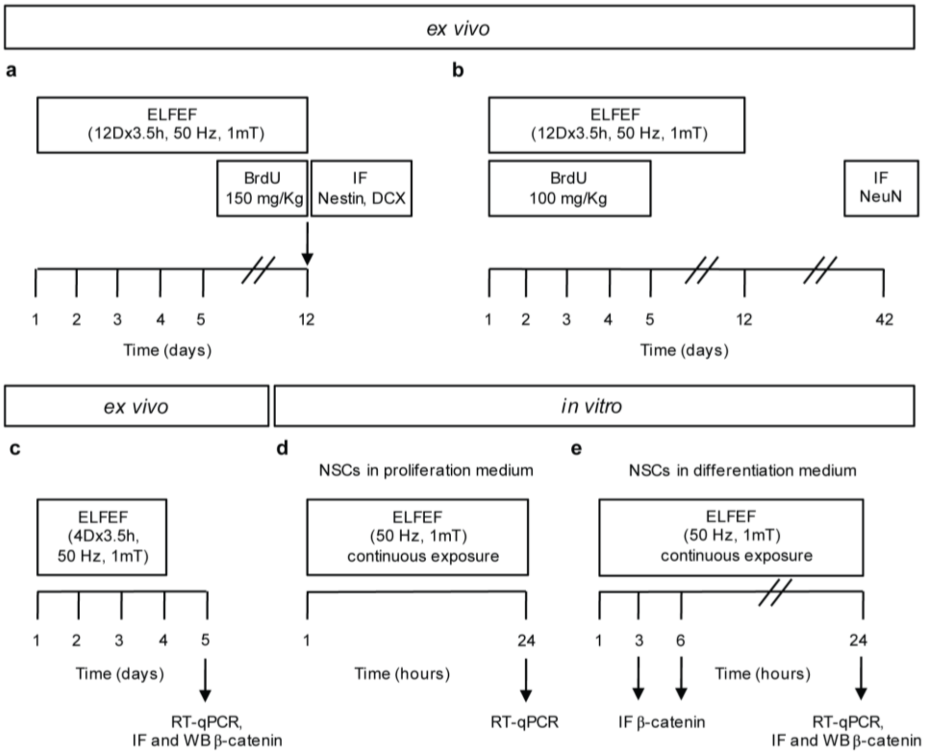

Supplementary Figure 8. Experimental design and timeline of the protocol.
